# Enhancing prime editor flexibility with coiled-coil heterodimers

**DOI:** 10.1186/s13059-024-03257-z

**Published:** 2024-04-26

**Authors:** Shuangshuang Mu, Huangyao Chen, Qianru Li, Shixue Gou, Xiaoyi Liu, Junwei Wang, Wei Zheng, Menglong Chen, Qin Jin, Liangxue Lai, Kepin Wang, Hui Shi

**Affiliations:** 1 China-New Zealand Joint Laboratory On Biomedicine and Health, CAS Key Laboratory of Regenerative Biology, Joint School of Life Sciences, Guangzhou Institutes of Biomedicine and Health, Chinese Academy of Sciences, Guangzhou Medical University, Guangzhou, 510530 China; 2 Guangdong Provincial Key Laboratory of Stem Cell and Regenerative Medicine, Centre for Regenerative Medicine and Health, Hong Kong Institute of Science and Innovation, Guangzhou Institutes of Biomedicine and Health, Chinese Academy of Sciences, Guangzhou, 510530 China; 3 University of Chinese Academy of Sciences, Beijing, 100049 China; 4 Hainan Provincial Research Centre of Laboratory Animals, Sanya Institute of Swine Resource, Sanya, 572000 China; 5 Research Unit of Generation of Large Animal Disease Models, Chinese Academy of Medical Sciences (2019RU015), Guangzhou, 510530 China; 6 Guangzhou National Laboratory, Guangzhou, 510005 China; 7 Guangdong Provincial Key Laboratory of Large Animal Models for Biomedicine, Wuyi University, Jiangmen, 529020 China; 8 Department of Neurology and Stroke Centre, The First Affiliated Hospital, Jinan University, Guangzhou, 510630 China

## Abstract

**Background:**

Prime editing enables precise base substitutions, insertions, and deletions at targeted sites without the involvement of double-strand DNA breaks or exogenous donor DNA templates. However, the large size of prime editors (PEs) hampers their delivery in vivo via adeno-associated virus (AAV) due to the viral packaging limit. Previously reported split PE versions provide a size reduction, but they require intricate engineering and potentially compromise editing efficiency.

**Results:**

Herein, we present a simplified split PE named as CC-PE, created through non-covalent recruitment of reverse transcriptase to the Cas9 nickase via coiled-coil heterodimers, which are widely used in protein design due to their modularity and well-understood sequence-structure relationship. We demonstrate that the CC-PE maintains or even surpasses the efficiency of unsplit PE in installing intended edits, with no increase in the levels of undesired byproducts within tested loci amongst a variety of cell types (HEK293T, A549, HCT116, and U2OS). Furthermore, coiled-coil heterodimers are used to engineer SpCas9-NG-PE and SpRY-PE, two Cas9 variants with more flexible editing scope. Similarly, the resulting NG-CC-PE and SpRY-CC-PE also achieve equivalent or enhanced efficiency of precise editing compared to the intact PE. When the dual AAV vectors carrying CC-PE are delivered into mice to target the *Pcsk9* gene in the liver, CC-PE enables highly efficient precise editing, resulting in a significant reduction of plasma low-density lipoprotein cholesterol and total cholesterol.

**Conclusions:**

Our innovative, modular system enhances flexibility, thus potentially facilitating the in vivo applicability of prime editing.

**Supplementary Information:**

The online version contains supplementary material available at 10.1186/s13059-024-03257-z.

## Background

**Fig. 1:**
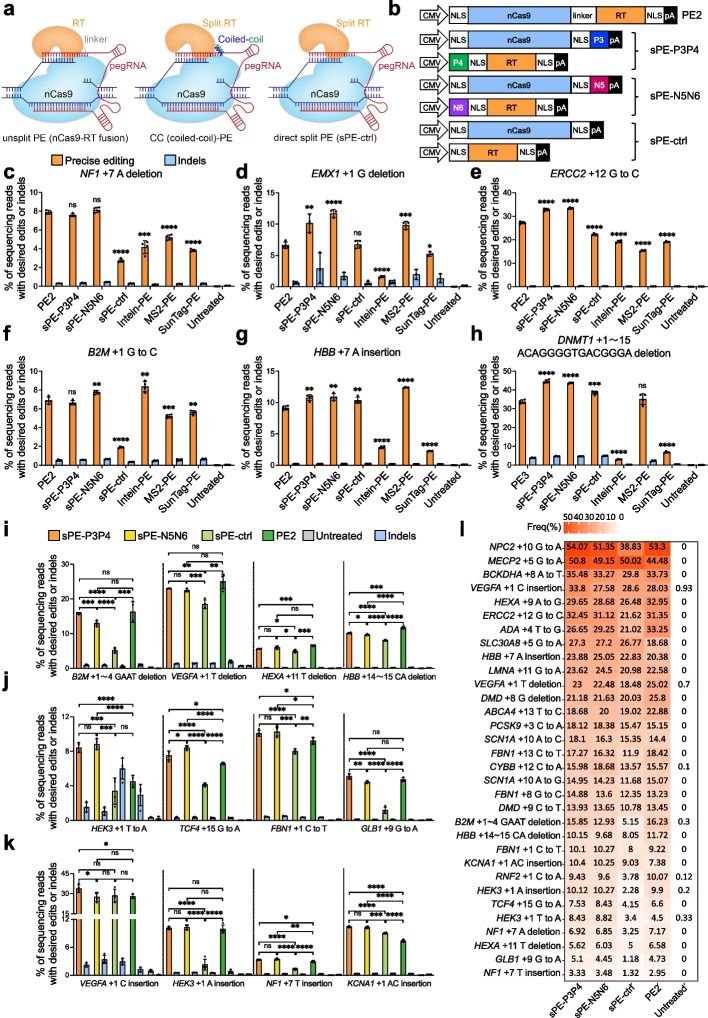
CC-PE enables precise genome editing at multiple endogenous sites in HEK293T cells. **a** Schematic diagrams of PE2, CC-PE, and direct split PE. PE2 consists of nCas9 (H840A), M-MLV RT and a pegRNA. CC-PE is expressed from two separate vectors with coiled-coil heterodimer. **b** Schematics of PE2, CC-PE, and direct split PE. **c**-**h** Comparison of the efficiencies of prime edits and indel byproducts generated by unsplit PE, sPE-P3P4, sPE-N5N6, sPE-ctrl, Intein-PE, MS2-PE, and SunTag-PE at *NF1* (**c**), *EMX1* (**d**), *ERCC2* (**e**), *B2M* (**f**), *HBB* (**g**), and *DNMT1* (**h**) loci in HEK293T cells. **i** Precise base deletion and undesired indel efficiencies of sPE-P3P4, sPE-N5N6, sPE-ctrl, and PE2 revealed by high-throughput sequencing among 4 tested loci. **j** Precise base substitution and undesired indel efficiencies of sPE-P3P4, sPE-N5N6, sPE-ctrl, and PE2 among 4 tested loci. **k** Precise base insertion and undesired indel efficiencies of sPE-P3P4, sPE-N5N6, sPE-ctrl, and PE2 among 4 tested loci. **l** The heatmap generated by high-throughput sequencing shows average precise editing efficiencies of sPE-P3P4, sPE-N5N6, sPE-ctrl, and PE2 among 32 tested loci. Statistical significance in **c**-**k** was determined via unpaired *t*-tests (*P < 0.05, **P < 0.01, ***P < 0.001, ****P < 0.0001, ns indicating not significant). Error bars indicated the mean ± standard deviation of at least three independent biological replicates

The demand for programmable introduction of desired small insertions, small deletions, and any base substitutions of four nucleotides into genome has driven the development of prime editing. The technique is based on the fusion of a Cas9 nickase (nCas9) and an engineered Moloney murine leukemia virus (M-MLV) reverse transcriptase (RT), in combination with a prime editing guide RNA (pegRNA), which contains a sgRNA, as well as a 3′ extension encoding the reverse transcription template (RTT) and the primer binding site (PBS) (Fig. 1a) [1]. Despite its versatility and precision in genome editing, the excessive size of current prime editors (PEs) hampers efficient delivery in vivo. Adeno-associated virus (AAV) is considered as one of the most appropriate vehicles to deliver the PE system to target organs/tissues for in vivo gene therapy given its low immunogenicity, broad tropism, and ease of production [2]. Yet, the large size of PE cassette is beyond the packaging capacity of AAV. Several strategies, including split inteins, MS2, SunTag, and the direct split PE (sPE) system, have been explored to solve this issue [3–8]. However, these strategies have their own limitations. Intein-based split PE system relies on intein-mediated trans-splicing to reconstitute the split nCas9 [9]. Nevertheless, the process of selecting the split site is laborious and impacts prime editing efficiency [5, 7]. Both MS2-PE and SunTag-PE systems are complicated, reflected by the fact that MS2 requires special sgRNA engineering and SunTag requires multiple GCN4 repeats. Moreover, the editing efficiencies of MS2- and SunTag-tethered configurations are not comparable to the unsplit PE to some extent [3]. In the direct split PE system, the nCas9 remains untethered from the RT (Fig. 1a), which had been claimed to function as efficiently as intact PE [3, 4]. However, in consideration of the absence of affinity modules, the binding of RT to the target genomic site is challenging. Theoretically, this issue could be overcome by using superior protein–protein affinity module to fuse RT with nCas9. Coiled-coil dimerization (CC) peptides enable tight and specific dimerization pairing based on electrostatic and hydrophobic interactions [10, 11], can serve as an alternative to reunite dimerization. Taking advantage of the rather simple structural architecture, we developed a CC-PE system that utilizes CC peptides to recruit RT to nCas9 in non-covalent manner (Fig. 1a). The effectiveness of CC peptides split system was validated in multiple PEs including PE2, PE3, NG-PE, and SpRY-PE through editing multiple endogenous loci in a variety of mammalian cell types. All 4 versions of CC-PEs demonstrated similar or improved efficiencies of precise editing without an increase in undesired byproducts compared to the unsplit PE in most of the tested loci. Furthermore, when the dual AAV vectors of CC-PE were delivered into the mouse liver to target *Pcsk9* gene, CC-PE system achieved precise editing with an efficiency as high as 45.9%, significantly reducing the levels of plasma low-density lipoprotein cholesterol and total cholesterol. Consequently, the CC-PE system exhibits considerable potential for therapeutic applications by providing a simple, flexible, and efficient method for in vivo gene editing.

## Results

### Design of CC-PE and comparative analysis with existing split strategies

To facilitate the flexibility of PE system, nCas9 and RT were fused to previously described coiled-coil dimer-forming peptides, P3-P4 pair [12] or N5-N6 pair [13], allowing the nCas9 and RT to dimerize at the target site (Fig. 1a). To construct CC-PE, we fused P3 or N5 to the C terminus of nCas9 and attached P4 or N6 to the N terminus of RT (Fig. 1b). As a result, P4-RT and N6-RT were recruited to nCas9-P3 and nCas9-N5, respectively. We designated nCas9-P3/P4-RT and nCas9-N5/N6-RT as sPE-P3P4 and sPE-N5N6, respectively (Fig. 1b). We compared editing efficiency of CC-PE with unsplit PE and four previously reported split strategies, including direct split PE (sPE) without affinity modules (sPE-ctrl), Intein-PE, MS2-PE, and SunTag-PE, across six endogenous loci (*NF1*, *EMX1*, *ERCC2*, *B2M*, *HBB*, and *DNMT1*) in HEK293T cells through deep amplicon sequencing. The results showed that CC-PE had increased, or at least, equivalent precise editing efficiencies compared to those of unsplit PE, sPE, Intein-PE, MS2-PE, and SunTag-PE at all six tested endogenous loci (Fig. 1c-h; Additional file 1: Fig. S1). We also noted that sPE-ctrl achieved higher or comparable efficiencies compared to the other split systems of PE (Additional file 1: Fig. S1), thus we chose sPE-ctrl as the control for subsequent studies. Notably, we found that the efficiencies of CC-PE-mediated precise editing were higher than those of sPE-ctrl at all tested endogenous sites and unsplit PE at four endogenous sites (*EMX1*, *ERCC2*, *HBB*, and *DNMT1*) in HEK293T cells (Fig. 1c-h).

To proceed to a broader scale, next, we further performed comparative analysis of the editing efficiencies of CC-PE, sPE-ctrl, and unsplit PE2 at 29 endogenous loci in human HEK293T cells. We designed pegRNAs for insertions at *VEGFA*, *HEK3*, *NF1*, and *KCNA1* genes; deletions at *B2M*, *VEGFA*, *HEXA*, *HBB*, and *DMD* genes; and various base substitutions at *NPC2*, *MECP2*, *BCKDHA*, *HEXA*, *ADA*, *SLC30A8*, *LMNA*, *ABCA4*, *PCSK9*, *SCN1A*, *FBN1*, *CYBB*, *DMD*, *RNF2*, *TCF4*, *HEK3*, and *GLB1* genes (Fig. 1i-l; Additional file 1: Fig. S2). Compared to sPE-ctrl (1.18% to 50.02%), deep amplicon sequencing revealed that precise editing efficiencies of CC-PE (sPE-P3P4, 3.33% to 54.07%; and sPE-N5N6, 3.48% to 51.35%) were significantly improved at most of the genomic sites (25 out of 29, 86.2%) (Fig. 1i-l; Additional file 1: Fig. S2). The maximum performance improvements achieved were 4.44-fold by sPE-P3P4 and 4.50-fold by sPE-N5N6 at the *HEK3* locus for A insertion (Fig. 1k-l). For four remaining genomic sites (*VEGFA* for C insertion, *ABCA4* for T-to-C substitution, *SLC30A8* for G-to-A substitution, and *MECP2* for G-to-A substitution), CC-PE achieved comparable levels of precise editing to sPE-ctrl (Fig. 1k-l; Additional file 1: Fig. S2o-q). Additionally, sPE-P3P4 or sPE-N5N6 demonstrated comparable in 8 out of 29 loci (27.6%), and even significantly higher efficiencies in 14 out of 29 loci (48.3%), compared to unsplit PE2 at the tested loci (Fig. 1i-l; Additional file 1: Fig. S2). Thus, these data suggest that the editing efficiency of CC-PE surpasses sPE-ctrl, while matching or exceeding that of PE2.

### CC-PE exhibits similar frequency of undesired indel byproducts to unsplit PE2

We next evaluated whether CC-PE would result in an undesired indel formation surge, which is associated with safety concerns in gene therapy using this system. High-throughput sequencing showed that across all tested genomic loci, the undesired indel byproduct proportion generated by CC-PE was not elevated compared to both sPE-ctrl and unsplit PE2 (Additional file 1: Fig. S3). More detailly, we assessed the indel patterns and frequencies at three genomic sites, *HEK3* (+ 1 T to A), *B2M* (+ 1 GAAT deletion), and *VEGFA* (+ 1 C insertion). The indel frequencies of sPE-P3P4 (1.04%) and sPE-N5N6 (1.20%) were lower than those of unsplit PE2 (3.73%) and sPE-ctrl (4.50%) at the *HEK3* (+ 1 T to A) locus, representing more than threefold reduction (Additional file 1: Fig. S4a), and the indel frequencies of the CC-PE at the *B2M* (+ 1 GAAT deletion) and *VEGFA* (+ 1 C insertion) loci were similar to those of unsplit PE2 and sPE-ctrl (Additional file 1: Fig. S5a, S6a). We also further analyzed the concrete undesired insertion and deletion frequencies generated by CC-PE, PE2, and sPE-ctrl. Consistently, the frequencies of undesired insertions (Additional file 1: Fig. S4b-e) and deletions (Additional file 1: Fig. S4f-i) of CC-PE were lower than those of unsplit PE2 at the *HEK3* (+ 1 T to A) locus. In addition, at the *B2M* (+ 1 GAAT deletion) and *VEGFA* (+ 1 C insertion) loci, the undesired insertions (Additional file 1: Fig. S5b-e, S6b-e) and deletions (Additional file 1: Fig. S5f-i, S6f-i) produced by CC-PE were similar to unsplit PE2 and sPE-ctrl. These data suggest that CC-PE does not result in excessive undesired indel byproducts, conferring CC-PE with the potential for therapeutic application.

### Evaluation of performance of the coiled-coil heterodimer-mediated split strategy on the PE3 editor

**Fig. 2:**
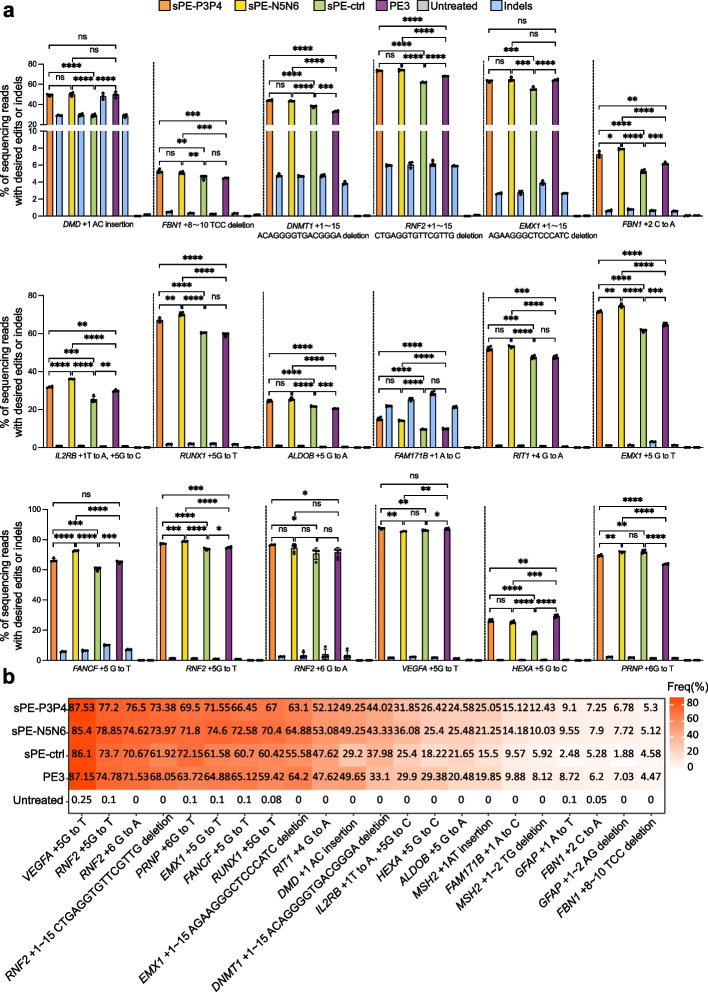
CC-PE combining with the PE3 strategy enables precise genome editing. **a** Precise editing and undesired indel efficiencies of PE3, sPE-P3P4, sPE-N5N6, and sPE-ctrl at *DMD* (+ 1 AC insertion), *FBN1* (+ 8 ~ 10 TCC deletion), *DNMT1* (+ 1 ~ 15 ACAGGGGTGACGGGA deletion), *RNF2* (+ 1 ~ 15 CTGAGGTGTTCGTTG deletion), *EMX1* (+ 1 ~ 15 AGAAGGGCTCCCATC deletion), *FBN1* (+ 2 C to A), *IL2RB* (+ 1 T to A, + 5 G to C), *RUNX1* (+ 5 G to T), *ALDOB* (+ 5 G to A), *FAM171B* (+ 1 A to C), *RIT1* (+ 4 G to A), *EMX1* (+ 5 G to T), *FANCF* (+ 5 G to T), *RNF2* (+ 5 G to T), *RNF2* (+ 6 G to A), *VEGFA* (+ 5 G to T), *HEXA* (+ 5 G to C), and *PRNP* (+ 6 G to T) loci. **b** The heatmap generated by high-throughput sequencing shows average precise editing efficiencies of PE3, sPE-P3P4, sPE-N5N6, and sPE-ctrl among the 22 tested loci. Statistical significance in **a** was determined via unpaired *t*-tests (*P < 0.05, **P < 0.01, ***P < 0.001, ****P < 0.0001, ns indicating not significant). Error bars indicated the mean ± standard deviation of at least three independent biological replicates

Next, we also evaluated the performance of the coiled-coil heterodimer-mediated split strategy on the PE3 editor, an optimized strategy being able to improve prime editing efficiency, which employs an additional sgRNA to nick the non-edited strand using the Cas9(H840A) nickase already present in PE2, directing DNA repair by using the edited strand as the template [1]. To assess the effectiveness of CC-PE editor with PE3 strategy, we examined frequencies of various types of precise edits including insertions, deletions, and substitutions in 22 genomic loci via deep amplicon sequencing. The precise editing efficiencies of CC-PE among these loci ranged from 5.12% to 87.53%, exceeding 50% in 10 genomic loci. (Fig. 2a, b). The sPE-P3P4 and sPE-N5N6 resulted in significantly higher efficiencies of precise edits than those of direct split PE at all tested loci except for *RNF2* (+ 6 G to A), *VEGFA* (+ 5 G to T), and *PRNP* (+ 6 G to T) loci (Fig. 2a; Additional file 1: Fig. S7). Additionally, they displayed significantly higher efficiencies at 14 out of 22 (63.6%) loci and showed no significant difference at 7 out of 22 (31.8%) loci when compared to unsplit PE3 (Fig. 2a; Additional file 1: Fig. S7). The sPE-P3P4 and sPE-N5N6 exhibited stronger precise editing activities than both unsplit PE3 and sPE-ctrl at 13 out of 22 (59.1%) tested loci (Fig. 2a; Additional file 1: Fig. S7). Specifically, as an example, we noted that when the *DMD* locus, a therapeutically relevant gene, was used as the gene of interest, the precise insertion efficiencies of both sPE-P3P4 and sPE-N5N6 were comparable to intact PE3, while significantly superior to that of sPE-ctrl (Fig. 2a; Additional file 1: Fig. S8). These results reaffirmed that CC-PE editor outperforms direct split PE when combined with the PE3 strategy, while maintaining or exceeding the capabilities of unsplit PE3.

### Efficient precise editing by CC-PE in multiple mammalian cell lines

**Fig. 3:**
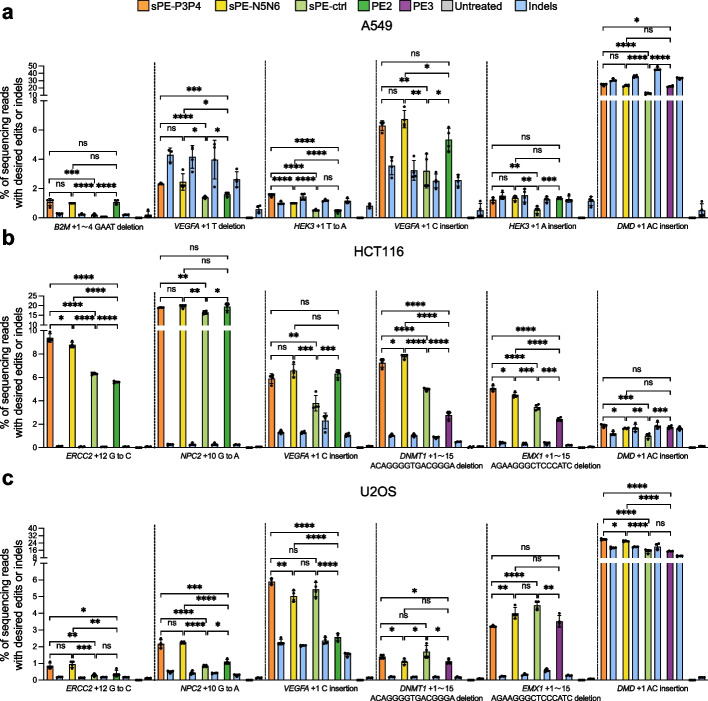
CC-PE performs prime editing in multiple mammalian cell lines. **a** Prime editing with sPE-P3P4, sPE-N5N6, sPE-ctrl, and unsplit PE in A549 cells. Nick gRNA was included in each PE system at *DMD* (+ 1 AC insertion). **b** Prime editing with sPE-P3P4, sPE-N5N6, sPE-ctrl, and unsplit PE in HCT116 cells. Nick gRNA was included in each PE system at *DNMT1* (+ 1 ~ 15 ACAGGGGTGACGGGA deletion), *EMX1* (+ 1 ~ 15 AGAAGGGCTCCCATC deletion), and *DMD* (+ 1 AC insertion). **c** Prime editing with sPE-P3P4, sPE-N5N6, sPE-ctrl, and unsplit PE in U2OS cells. Nick gRNA was included in each PE system at *DNMT1* (+ 1 ~ 15 ACAGGGGTGACGGGA deletion), *EMX1* (+ 1 ~ 15 AGAAGGGCTCCCATC deletion), and *DMD* (+ 1 AC insertion). Statistical significance in **a-c** was determined via unpaired *t*-tests (*P < 0.05, **P < 0.01, ***P < 0.001, ****P < 0.0001, ns indicating not significant). Error bars indicated the mean ± standard deviation of at least three independent biological replicates

The findings above showed that CC-PE had similar or even superior efficiencies compared to unsplit PE at various genomic loci in HEK293T cells. We next examined the universality of CC-PE in other human cell lines. The editing activities of sPE-P3P4, sPE-N5N6, sPE-ctrl, and unsplit PE were examined at six genomic loci in each of the three cell lines including A549, HCT116, and U2OS cells (Fig. 3a-c). In A549 cells, both sPE-P3P4 and sPE-N5N6 resulted in higher editing efficiencies compared to sPE-ctrl and similar efficiencies to intact PE at *B2M* (+ 1 ~ 4 GAAT deletion) and *HEK3* (+ 1 A insertion) loci (Fig. 3a). Additionally, significantly increased efficiencies of sPE-P3P4 or sPE-N5N6 compared to sPE-ctrl and unsplit PE were observed at *HEK3* (+ 1 T to A), *VEGFA* (+ 1 T deletion and + 1 C insertion), and *DMD* (+ 1 AC insertion) loci (Fig. 3a). In HCT116 cells, sPE-P3P4 and sPE-N5N6 resulted in higher editing efficiencies compared to sPE-ctrl and similar efficiencies to intact PE at *NPC2* (+ 10 G to A), *VEGFA* (+ 1 C insertion), and *DMD* (+ 1 AC insertion) loci (Fig. 3b). The efficiencies of sPE-P3P4 and sPE-N5N6 were significantly higher than those of sPE-ctrl and unsplit PE at *ERCC2* (+ 12 G to C), *DNMT1* (+ 1 ~ 15 ACAGGGGTGACGGGA deletion), and *EMX1* (+ 1 ~ 15 AGAAGGGCTCCCATC deletion) (Fig. 3b). In U2OS cells, the sPE-P3P4 and sPE-N5N6 achieved significantly increased efficiencies compared to sPE-ctrl and unsplit PE at *ERCC2* (+ 12 G to C), *NPC2* (+ 10 G to A), and *DMD* (+ 1 AC insertion) loci (Fig. 3c). Both sPE-P3P4 and sPE-N5N6 exhibited significantly increased efficiencies compared to unsplit PE and similar efficiencies to sPE-ctrl at *VEGFA* (+ 1 C insertion) locus (Fig. 3c). In addition to the above loci, similar editing efficiencies of CC-PE, sPE-ctrl, and unsplit PE were observed at *EMX1* (+ 1 ~ 15 AGAAGGGCTCCCATC deletion) and *DNMT1* (+ 1 ~ 15 ACAGGGGTGACGGGA deletion) loci (Fig. 3c).

In general, across all 18 edits at eight endogenous genes, both sPE-P3P4 and sPE-N5N6 exhibited superior efficiencies to sPE-ctrl at 15 edits and demonstrated similar or enhanced precise editing activities than those of unsplit PE across all tested loci. These data suggest that the editing efficiency of CC-PE typically surpasses that of direct split PE system and is comparable or even superior to that of intact PE system across various cell lines.

### Engineering SpCas9-NG-PE and SpRY-PE with coiled-coil heterodimers for more flexible editing scope

Conventionally, the targetable regions within the genome using conventional prime editors are greatly limited due to the NGG PAM preference of SpCas9. Subsequent reports showed that integrating evolved Cas9 variants, such as SpCas9-NG with NG PAM and SpCas9-SpRY with NRN PAM, into prime editors conferred them with the capacity to expand the scope of genome editing [14, 15]. Therefore, we proceeded to design two additional types of CC-PE with more flexible PAM: NG-CC-PE (NG-sPE-P3P4 and NG-sPE-N5N6) and SpRY-CC-PE (SpRY-sPE-P3P4 and SpRY-sPE-N5N6), respectively. Accordingly, the pegRNAs were designed to bear NG PAM or NRN PAM, which could introduce diverse types of mutations, including insertions, deletions, and substitutions at target sites.

**Fig. 4:**
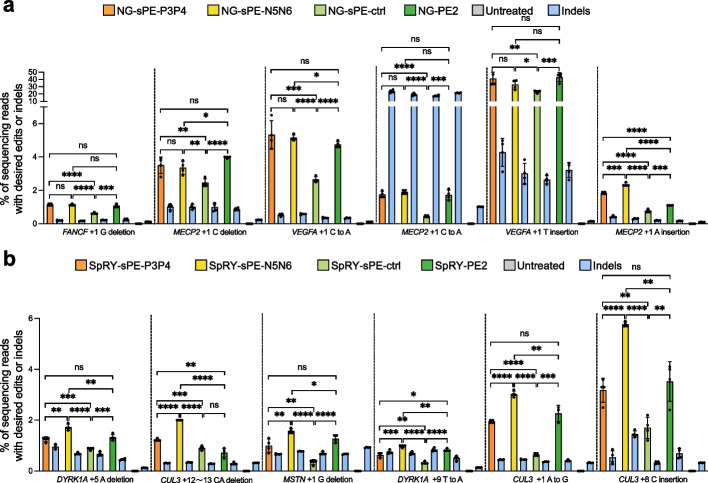
Prime editing using NG-CC-PE or SpRY-CC-PE system. **a** Precise editing and undesired indel efficiencies of NG-sPE-P3P4, NG-sPE-N5N6, NG-sPE-ctrl, and NG-PE2 at *FANCF* (+ 1 G deletion), *MECP2* (+ 1 C deletion), *VEGFA* (+ 1 C to A), *MECP2* (+ 1 C to A), *VEGFA* (+ 1 T insertion), and *MECP2* (+ 1 A insertion) loci in HEK293T cells. **b** Precise editing and undesired indel efficiencies of SpRY-sPE-P3P4, SpRY-sPE-N5N6, SpRY-sPE-ctrl, and SpRY-PE2 at *DYRK1A* (+ 5 A deletion), *CUL3* (+ 12 ~ 13 CA deletion), *MSTN* (+ 1 G deletion), *DYRK1A* (+ 9 T to A), *CUL3* (+ 1 A to G), and *CUL3* (+ 8 C insertion) loci in HEK293T cells. Data in **a** and **b** were obtained from three or more independent biological replicates (mean ± s.d.) via unpaired *t*-tests (*P < 0.05, **P < 0.01, ***P < 0.001, ****P < 0.0001, ns indicating not significant)

For SpCas9-NG, a variant that can expand the targeting range by relaxing preferences for the third nucleobase of the PAM [16], we examined the editing efficiencies at 9 NG PAM target sites using the NG-CC-PE system. Increased precise editing efficiencies of NG-sPE-P3P4 and NG-sPE-N5N6 were obtained compared to NG-sPE-ctrl at all tested loci except for *FANCF* (+ 1 C to A) (Fig. 4a; Additional file 1: Fig. S9a-c). The precise editing efficiencies of NG-sPE-P3P4 or NG-sPE-N5N6 were comparable to NG-PE2 performance levels at *FANCF* (+ 1 G deletion), *MECP2* (+ 1 C deletion), *MECP2* (+ 1 C to A), and *VEGFA* (+ 1 T insertion) loci (Fig. 4a). Notably, at *VEGFA* (+ 1 C to A), *MECP2* (+ 1 A insertion), *FANCF* (+ 1 C to A), and *MECP2* (+ 1 C insertion) loci, NG-sPE-P3P4 or NG-sPE-N5N6 had higher average editing efficiencies than NG-PE2 (Fig. 4a; Additional file 1: Fig. S9a, b). Slightly lower performance of the NG-sPE-P3P4 and NG-sPE-N5N6 compared to NG-PE2 was observed at only one locus (*FANCF*, + 2 T insertion) (Additional file 1: Fig. S9c).

For SpCas9-SpRY, a variant almost entirely removing PAM restriction for genome editing [17], we designed SpRY-CC-PE system and tested its editing efficiency at 8 NRN PAM target loci. The SpRY-sPE-P3P4 and SpRY-sPE-N5N6 resulted in higher efficiencies of precise editing than that of SpRY-sPE-ctrl at all tested loci except for *MSTN* (+ 4 CT insertion) locus (Fig. 4b; Additional file 1: Fig. S9d, e). The editing efficiency of SpRY-sPE-P3P4 was comparable to that of SpRY-PE2, while SpRY-sPE-N5N6 outperformed SpRY-PE2 at *DYRK1A* (+ 5 A deletion), *MSTN* (+ 1 G deletion), *CUL3* (+ 1 A to G), *CUL3* (+ 8 C insertion), *MSTN* (+ 14 T to G), and *MSTN* (+ 4 CT insertion) loci (Fig. 4b; Additional file 1: Fig. S9d, e). Notably, SpRY-sPE-P3P4 and SpRY-sPE-N5N6 achieved higher editing efficiencies than SpRY-PE2 at *CUL3* (+ 12 ~ 13 CA deletion) locus (Fig. 4b).

Overall, across different edits at endogenous genomic loci, NG-CC-PE and SpRY-CC-PE systems outperformed sPE-ctrl (15 of 17 loci, 88.2%) and achieved higher or similar editing efficiencies than unsplit PE2 (16 of 17 loci, 94.1%). These findings demonstrate that integrating previously identified SpCas9 variants into the CC-PE editor can relax conventional PAM preferences, thereby expanding the scope of spacer sequence selections and potential applications of CC-PE with PAM flexibility.

### Bioinformatic analysis of pegRNA-independent off-target effects of CC-PE through whole-genome sequencing

**Fig. 5:**
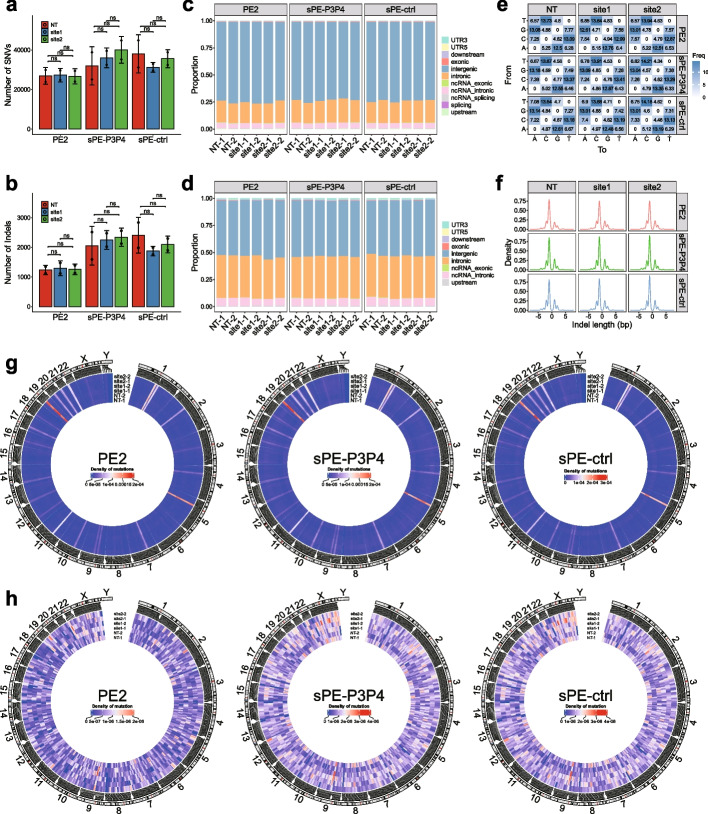
Whole genome sequencing analysis of HEK293T cells treated with sPE-P3P4, PE2 and sPE-ctrl. **a**-**b** Bar chart of the number of SNVs (**a**) and indels (**b**), data values represent the means, error bars correspond to SE, *n* = 2, unpaired *t*-test. **c**-**d** Bar chart of the distribution proportion of SNVs (**c**) and indels (**d**) on genomic features. **e** Heat map of the frequency of SNV base substitution. **f** Density plot of the distribution of indel length. **g**-**h** Circos plot of the distribution of SNVs (**g**) and indels (**h**) on the genome, color represents mutation density within the 1 × 10^7^ bp windows

Next, we performed whole-genome sequencing (WGS) on HEK293T cells to assess pegRNA-independent off-target effects of CC-PE at the genome-wide level. Due to the comparable precise editing efficiencies of sPE-P3P4 and sPE-N5N6 at all tested loci, sPE-P3P4 was selected as the representative for further off-target analysis. First, we assessed whether more mutations would be generated with the pegRNA of target site (site1, *B2M* + 1 ~ 4 deletion; site2, *HEK3* + 1 T to A) than those of the pegRNA of non-target site (NT). WGS results revealed that there was no significant difference in the number of single nucleotide variants (SNVs) and indels between the pegRNA of non-targeted site and the pegRNA of targeted site in sPE-P3P4, PE2, and sPE-ctrl (Fig. 5a, b). The Venn diagram displayed that very few overlapping mutations occurred across experimental samples and replicates, suggesting that these mutations were spontaneous events and happened randomly in the genome (Additional file 1: Fig. S10a, c).

We also examined the potential impact of these mutations on gene function. The results showed that there was no difference in the distribution of SNVs and indels on genomic features among samples (Fig. 5c, d). Only a small number of mutations resided within exons, and the count of these exon mutations did not present any significant difference across samples (Additional file 1: Fig. S11a, b). Furthermore, base substitution types of SNVs and lengths of indels were unbiased among samples (Fig. 5e, f; Additional file 1: Fig. S10b, d). For SNVs, the changes of amino acids resulting from mutations showed no difference among samples (Additional file 1: Fig. S11c). The mutations on genome-wide coordinates revealed by circos plots exhibited that most SNVs were distributed in mutation hotspots, and there were no regional and sample bias in SNVs and indels at the genome-wide level and exon level (Fig. 5g, h; Additional file 1: Fig. S11d). Taken together, these results suggest that these mutations happened spontaneously and randomly, and there is no extra pegRNA-independent off-target caused by CC-PE. This implies that CC-PE could be a practical genome editing tool with manageable mutation handling.

### Delivery of CC-PE by dual AAVs for in vivo genome editing

We next verified whether CC-PE could be delivered flexibly and yield precise editing in vivo using dual AAVs. To maximize in vivo prime editing efficiency, we applied the RNA structural motif, evopreQ_1_, to modify the 3′ terminus of pegRNAs (EpegRNA), a method that had been reported to improve prime editing efficiency by enhancing pegRNA stability and preventing its degradation [18].

**Fig. 6:**
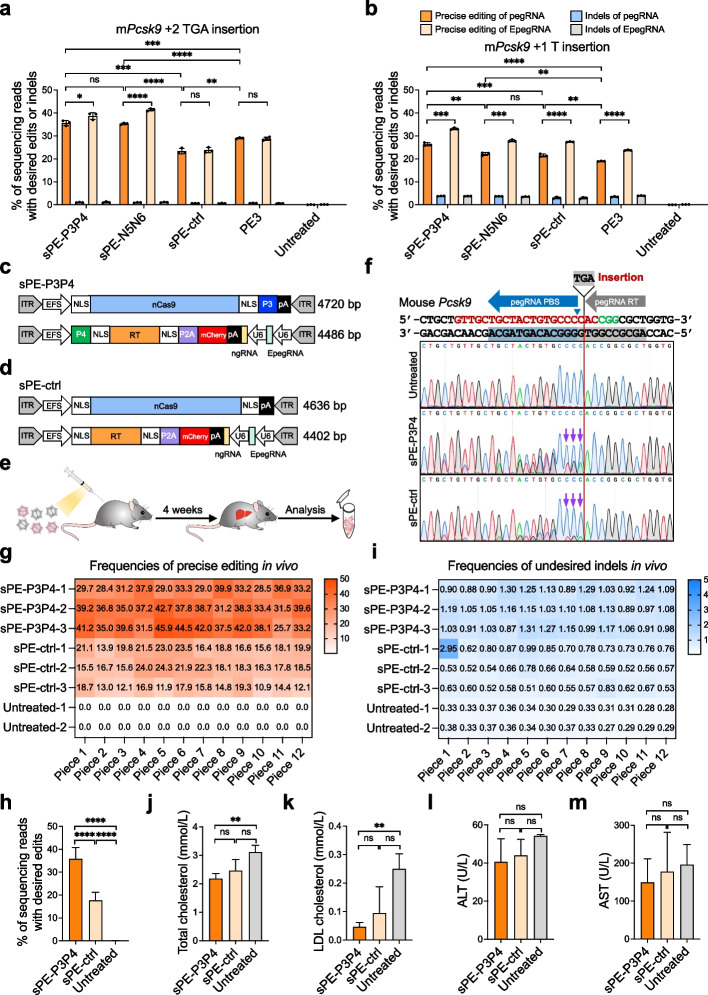
In vivo prime editing to *Pcsk9* and its effect on plasma cholesterol levels. **a**-**b**. Amplicon sequencing results showing prime editing efficiencies of sPE-P3P4, sPE-N5N6, sPE-ctrl, and PE3 at *Pcsk9* in MEFs. **c**-**d**. Schematics of dual-AAV sPE-P3P4 (**c**) and dual-AAV sPE-ctrl (**d**) architecture. **e** Schematic of in vivo prime editing. The dual-AAV sPE-P3P4 (*n* = 3) or dual-AAV sPE-ctrl (*n* = 3) was delivered to 2-week-old mice via intraperitoneal injection. **f** Sanger-sequencing chromatograms of sPE-P3P4 and sPE-ctrl harboring the desired insertion at Pcsk9 (+ 2 TAG insertion) locus in mouse livers. The purple arrow denotes the desired insertion. The *Pcsk9*-specific sgRNA is indicated in red and the PAM sequence is indicated in green. **g**-**i** Prime editing (**g, h**) and indel efficiencies (**i**) in bulk liver of sPE-P3P4 and sPE-ctrl after injection of AAV to 2-week-old mice (sPE-P3P4, *n* = 3; sPE-ctrl, *n* = 3; untreated, *n* = 2) via intraperitoneal injection (5 × 10^11^ viral genomes each). **j** Quantification of total plasma cholesterol levels at 4 weeks after injection. **k** Quantification of total plasma LDL cholesterol levels at 4 weeks after injection. **l** Plasma ALT was quantified in noninjected mice and AAV-injected mice. **m** Plasma AST was quantified in noninjected mice and AAV-injected mice. Statistical significance was determined via unpaired *t*-tests (*P < 0.05, **P < 0.01, ***P < 0.001, ****P < 0.0001, ns indicating not significant)

Murine proprotein convertase subtilisin/kexin type 9 (*Pcsk9*) was chosen as target gene for in vivo verification, due to its potential in treating familial hypercholesterolemia. We designed pegRNAs along with corresponding nicking sgRNAs targeting the first exon of *Pcsk9* gene to insert a stop codon (TGA) or T to abolish Pcsk9 function. Precise editing efficiencies were initially evaluated in vitro. Mouse embryonic fibroblasts (MEFs) were co-electroporated with the indicating pegRNA and unsplit/split PE3. Deep sequencing showed that sPE-P3P4 mediated precise insertion efficiencies were significantly higher compared to direct split PE (for TGA insertion: 35.6 ± 1.1 vs 23.4 ± 1.2; for T insertion: 26.3 ± 0.7 vs 21.5 ± 0.6) and unsplit PE3 (for TGA insertion: 35.6 ± 1.1 vs 29.0 ± 0.3; for T insertion: 26.3 ± 0.7 vs 19.0 ± 0.1) (Fig. 6a, b). The efficiencies of TGA insertion were overall higher than those of T insertion (Fig. 6a, b). We also observed that the modified EpegRNA possessed higher prime editing activity (Fig. 6a, b). Therefore, sPE-P3P4 and the EpegRNA with TGA insertion were chosen for package into AAV8, which exhibits high affinity for hepatocytes and specifically targets the liver, for disruption of *Pcsk9* gene in vivo (Fig. 6c). Two AAV vectors were constructed: the first vector contained EF-1a short promoter (EFS)-driven nCas9(H840A) tethered with coiled-coil peptide P3 at C-terminal; the second vector contained EFS-driven RT tethered with coiled-coil peptide P4 at N-terminal, U6 promoter-directed *Pcsk9*-EpegRNA, and corresponding nicking sgRNA expression cassettes (Fig. 6c). AAV vectors of the direct split PE system were also constructed as the control (Fig. 6d). All vectors were within the 4.8 kb packaging capacity of AAV and were packaged into AAV8 (Fig. 6c, d). The dual AAV8 sPE-P3P4 or sPE-ctrl (5 × 10^11^ viral genomes each) were administrated into 2-week-old mice via intraperitoneal injection (*n* = 3 for each group) (Fig. 6e). Four weeks after injection, total plasma and livers were harvested from injected and untreated control mice (Fig. 6e). Twelve pieces of samples were collected from the liver of each AAV8-injected (*n* = 3 for each group) or untreated (*n* = 2) control mice, and genomic DNA was extracted for further analysis. Sanger sequencing showed that sPE-P3P4 mediated higher prime editing efficiency compared to sPE-ctrl in vivo (Fig. 6f). Amplicon deep sequencing further confirmed the successful insertion of TGA stop codon into the *Pcsk9* gene in vivo by both sPE-P3P4 and sPE-ctrl (Fig. 6g). The average precise insertion efficiencies of sPE-P3P4 (35.8%, *n* = 3; ranging from 25.7% to 45.9%) significantly surpassed those of sPE-ctrl (17.6%, *n* = 3; ranging from 10.9% to 24.3%) (Fig. 6g, h). The average undesired indel efficiencies of sPE-P3P4 and sPE-ctrl were 1.1% (*n* = 3) and 0.7% (*n* = 3), respectively (Fig. 6i).

Loss of Pcsk9 leads to reduction in low-density lipoprotein (LDL) cholesterol [19]. Fully in line with Pcsk9 function, both total cholesterol and LDL cholesterol levels were significantly reduced in sPE-P3P4-injected mice compared to the untreated controls (Fig. 6j, k). Importantly, alanine aminotransferase (ALT) and aspartate transaminase (AST) levels in the injected mice were not elevated, suggesting no apparent hepatocellular injury (Fig. 6l, m). Altogether, these data robustly demonstrate that CC-PE can be effectively delivered for in vivo precise editing.

## Discussion

Due to the precise and versatile genome manipulation of PE in biomedical sciences, its higher efficiency and greater flexibility are highly desired. However, the full-length of PE2 exceeds the packaging capacity of AAV vectors, which poses a significant obstacle to in vivo delivery. To address this issue, several attempts have been performed to enhance the flexibility of PEs, including split inteins, MS2, SunTag, and direct split PE system, but these existing strategies are complicated, laborious, or compromise precise editing efficiency.

In this study, we present a flexible version of PE, termed CC-PE, where nCas9 and RT are coupled via coiled-coil dimerization peptides, which contain only 28 amino acids and nearly do not impose an additional burden of vector size. The construction of CC-PE is a much simpler process compared to previously reported split strategies, including split inteins, MS2, and SunTag. The feature of CC-mediated noncovalent affinity allows RT to specifically and tightly bind to nCas9 at the editing loci once they are translated into proteins. This provides an advantage over the direct split PE system, in which the untethered nCas9 and RT lack affinity and may not bind to each other. Therefore, our CC-PE system can achieve higher editing efficiencies across the majority of tested loci compared to the previously described direct split PE system, which is more efficient among all existing split strategies as confirmed in our testing system under the same conditions. We also observed that CC-PE not only functioned at the same level of editing efficiency but even outperformed unsplit PE in almost half of the tested loci (14 out of 29 for PE2; 14 out of 22 for PE3). The improvement in editing efficiency with CC-PE is theoretically feasible because a properly designed coiled-coil domain can provide a strong capacity for dimerizing two proteins [10, 11]. As a result, the tightly dimerized nCas9 and RT with the coiled-coil domain can enable more efficient collaborative editing compared to the original unsplit PE system, in which nCas9 remains loosely associated with RT through a linker.

CC-PE displays comprehensive universality in gene editing across various gene loci, cell lines, and SpCas9 variants. It has been proven to be effective in over 50 tested endogenous loci and 4 human cell lines, including HEK293T, A549, HCT116, and U2OS. Additionally, the incorporation of coiled-coil heterodimers into SpCas9-NG-PE and SpRY-PE, specifically designed for NG PAM and NRN PAM, respectively, makes the editing scopes more flexible, allowing for potential application of CC-PE with relaxed PAM compatibility. Furthermore, the rather simple modularity of coiled-coil dimerization peptides, consisting of only 28 amino acids, allows the strategy to split other gene editors, such as adenine base editor, cytidine base editor, dual base editor, and HDR stimulator, to reduce packaging size.

The safety concerns associated with gene therapy pose significant challenges, including potential issues such as increased undesired indel byproducts or off-target effects. In our system, high-throughput sequencing analysis revealed that the frequencies of undesired insertions and deletions generated by CC-PE were not higher compared to both sPE-ctrl and unsplit PE2 across all tested genomic loci. Furthermore, whole-genome sequencing demonstrated no significant difference in the number of SNVs and indels between non-targeted site pegRNA and targeted site pegRNA for CC-PE, PE2, and sPE-ctrl. The distribution of SNVs and indels on genomic features was also similar among the samples. These findings suggest that using the CC heterodimer as a split element to reduce the size of PEs did not result in additional off-target effects. The observed mutations are spontaneous events that routinely and randomly occur in any organism and may not have a potential negative impact on gene function. Therefore, CC-PE could be considered a genome editing tool as safe as unsplit PE and direct split PE for therapeutic applications. In practical application, when AAV-packaged CC-PE was delivered into mice to achieve precise edits at the *Pcsk9* locus, it enabled highly efficient prime editing in the mouse liver (up to 45.9%), which led to a significant reduction in plasma LDL cholesterol and total cholesterol, without any noticeable hepatocellular injury or toxicity.

## Conclusions

In summary, compared to unsplit PE and existing split PE strategies, including split inteins, MS2, SunTag, and the direct split PE, CC-PE established in this study offers a more simple, flexible, and effective approach for precise modification both in vitro and in vivo, especially for therapeutic applications.

## Materials and methods

### Plasmid construction

The CC-PE editor used in this study was constructed by pEASY-Uni Seamless Cloning and Assembly Kit (TransGen Biotech, CU101-02) using the pCMV-PE2 plasmid (Addgene, #132775) as the backbone. The P3 (GAGATCCAGCAGCTGGAGGAGGAGATCGCCCAGCTGGAGCAGAAGAACGCCGCCCTGAAGGAGAAGAACCAGGCCCTGAAGTAC), P4 (AAGATCGCCCAGCTGAAGCAGAAGATCGCCCAGCTGAAGCAGGAGAACCAGCAGCTGGAGGAGGAGAACGCCGCCCTGGAGTAC), N5 (GAGATCGCCGCCCTGGAGGCCAAGATCGCCGCCCTGAAGGCCAAGAACGCCGCCCTGAAGGCCGAGATCGCCGCCCTGGAGGCC), and N6 (AAGATCGCCGCCCTGAAGGCCGAGATCGCCGCCCTGGAGGCCGAGAACGCCGCCCTGGAGGCCAAGATCGCCGCCCTGAAGGCC) sequences were integrated into the vector by primer synthesis. In order to facilitate enrichment of transfected cells in subsequent experiments, we cloned PGK-mCherry and PGK-EGFP into the expression vectors of Cas9 and MLV, respectively.

To construct NG-CC-PE editor, the L1111R/D1135V/G1218R/E1219F/A1322R/R1335V/T1337R mutations were introduced into pCMV-PE2 vector. As for SpRY-CC-PE editor, the A61R/L1111R/D1135L/S1136W/G1218K/E1219Q/N1317R/A1322R/R1333P/R1335Q/T1337R mutations were integrated into pCMV-PE2 vector.

The inteins [20], MS2 [3], and SunTag [21] plasmids have been described previously. To construct the Cas9-NpuN plasmid, fragments of SpCas9 H840A (amino acid 1–713) and split-intein Npu N-terminal domain sequence were derived from Addgene #164909 via PCR. As for NpuC-Cas9-MLV plasmid, split-intein Npu C-terminal domain, SpCas9 H840A (amino acid 714–1368), and M-MLV RT from PE2 sequence were derived from Addgene #164908 via PCR. The MCP-MLV plasmid was constructed by replacing CC peptide with MCP stem loop aptamer. The MCP sequence was derived from Addgene #181799 via PCR. The Cas9-GCN4 plasmid was constructed by replacing CC peptide with 10 × GCN4. The 10 × GCN4 sequence was derived from Addgene #60903 via PCR. The scFv-MLV plasmid was constructed by replacing CC peptide with scFv fragment. The scFv sequence was derived from Addgene #60904 via PCR. The pegRNAs and nicking sgRNAs used in this study were constructed by introduction of the sequences in Additional file 2: Table S1-S4 into the U6-sgRNA backbone (Addgene, #48962). The primer sequences used in this study were synthesized by Guangzhou IGE Biotechnology. All vectors were confirmed by Sanger sequencing.

### Cell line authentication

HEK293T, A549, HCT116 or U2OS cell lines used in this study were obtained from the American Type Culture Collection (ATCC). These cell lines were authenticated by ATCC and not validated further in our laboratory. MEFs were isolated from wild type E13.5 embryos and validated by our laboratory.

### Cell culture and transfection

HEK293T, A549, HCT116, or U2OS cells were cultured in high-glucose Dulbecco’s modified Eagle’s medium (DMEM; HyClone) supplemented with 10% fetal bovine serum (FBS; Gibco), whereas the MEFs were cultured in DMEM supplemented with 15% FBS (Gibco), 1% nonessential amino acids (Gibco), 2 mM GlutaMAX (Gibco) and 1 mM sodium pyruvate (Gibco) at 37 °C and 5% CO_2_, with medium changes every day. When the cell confluency reached around 90%, cells were passaged and expanded using 0.25% trypsin. For HEK293T cells, prior to transfection, cells were digested into single cells and seeded into a 24-well plate. PEI transfection was performed when the cell confluency reached 40–60%. The medium is replaced with 2% FBS medium before transfection. PEI (Sigma, 408727; final concentration: 1 μg/μL) was gently mixed with 2% FBS medium and left at room temperature for 5 min. For each well, 3 μg of PEI and 25 μl of 2% FBS medium were mixed. Then, plasmids were added to the 2% FBS medium. For each well, about 1 or 1.125 μg of plasmid (250 ng of pegRNA + 750 ng of PE2 or sPE at 375 ng each for PE2 strategy, 125 ng of nicking sgRNA + 250 ng of pegRNA + 750 ng of PE2 or sPE at 375 ng each for PE3 strategy) and 25 μl of 2% FBS medium were mixed. The PEI mixture was then added dropwise into the plasmid mixture, gently mixed with a pipette, and left at room temperature for 20 min. The mixture of PEI and plasmid was then added dropwise to the HEK293T cells. 12 h after transfection, the cells were cultured with 10% FBS medium. For A549, U2OS and MEFs cells, cells were co-electrotransfected with 1 μg of pegRNA and 3 μg of PE2 or sPE (1.5 μg + 1.5 μg) for PE2 strategy, 0.5 μg of nicking sgRNA, 1 μg of pegRNA and 3 μg of PE2 or sPE (1.5 μg + 1.5 μg) for PE3 strategy at 1230 v, 30 ms, and 2 pulse (A549), 1230 v, 10 ms, and 4 pulse (U2OS), or 1350 v, 30 ms, and 1 pulse (MEFs) by using the Neon™ transfection system (Life Technology). For HCT116, cells were seeded in 24-well and transfected at approximately 50% confluence using Lipo8000 (Beyotime) according to the manufacturer’s protocols. For each well, 250 ng of pegRNA + 750 ng of PE2 or sPE at 375 ng of each for PE2 strategy, 125 ng of nicking sgRNA, 250 ng of pegRNA + 750 ng of PE2 or sPE at 375 ng each for PE3 strategy plasmids were used.

### FACS, cell lysis and PCR identification

Fluorescence-activated cell sorting was performed 72 h after transfection using MoFlo Astrios. The cells were collected after washing with PBS and digesting with 0.25% trypsin, then centrifuged at 200 g for 3 min and resuspended in PBS. For split PE, mCherry and EGFP double-positive cells were sorted, whereas for PE2, EGFP-positive cells were sorted, indicating the successful transfection of Cas9 and MLV expression vectors into the sorted cells. Approximately 10,000-50,000 sorted cells were subjected to further analysis. The sorted cells were lysed with NP40 following the condition: 56 °C for 60 min and 96 °C for 10 min. Subsequently, PCR amplification of the target site was performed using Q5 Hot Start High-Fidelity 2X Master Mix polymerase (NEB, M0494S).

### High-throughput sequencing and data analysis

For library preparation, amplification primers were designed near the target locus, with specific primers amplifying fragments within 190 bp. PE150 sequencing was typically used. Subsequently, two rounds of PCR amplification were performed. For the first round of PCR, amplification was performed using the primers listed in Additional file 2: Table S1-S4, with cell lysate as the template, and 30 cycles of amplification in a 25 μL reaction volume. For the second round of PCR, amplification was performed using primers with unique Illumina barcodes, with the first round PCR product as the template, and 12 cycles of amplification. The second round PCR products were purified using HiPure Gel Pure DNA Mini Kit (Magen, D2111-03) and quantified using Equalbit™ dsDNA HS Assay Kit (Vazyme) before subjecting to deep sequencing using Illumina HiSeq X platform (Annoroad Gene Technology Corporation). The high-throughput sequencing data were analyzed using PE-Analyzer (http://www.rgenome.net/pe-analyzer/) [22].

### Whole genome sequencing data analysis

Raw reads were processed by fastp (version 0.20.1) [23] to filter out low quality reads and remove sequencing adapters. Then, clean reads were aligned to the human genome defined by NCBI (GRCh38) with BWA (version 0.7.15-r1140) [24]. Aligned reads were recorded in bam format and processed by samtools (version 1.3.1) [25]. PCR duplicates were removed using sambamba (version 0.6.6) [26]. The software strelka2 (version 2.9.10) [27] was used to detect the somatic mutations in genome. The genomic information of SNVs and indels was annotated by ANNOVAR (version 2017Jul17) [28]. Venn plot was constructed using R package venn (verion 1.11), and circos plot of genomic mutation distribution was constructed using R package circlize (version 0.4.15).

### AAV production and injection

AAV8 viruses were packaged and produced by PackGene Biotech Co., Ltd. (Guangzhou, China). Before injection, the mice were anaesthetized by abdominal injection of 5% chloral hydrate. Dual AAV sPE-P3P4 (5 × 10^11^ genome copies of AAV-nCas9-P3 and 5 × 10^11^ genome copies of AAV-P4-MLV-mCherry-sgRNA-EpegRNA) or sPE-ctrl (5 × 10^11^ genome copies of AAV-nCas9 and 5 × 10^11^ genome copies of AAV-MLV-mCherry-sgRNA-EpegRNA) were delivered into 2 weeks old mice via intraperitoneal injection. The livers, predominantly dissociated into about 12 pieces, were harvested 4 weeks after AAV injection. The genomic DNA of liver samples was obtained by using TIANamp Genomic DNA Kit (TIANGEN).

### Blood biochemistry analysis

For blood biochemistry samples, mice were fasted overnight before blood collection via the ophthalmic vein. After standing at room temperature for 30 min, plasma was separated by centrifugation at 3000 rpm for 15 min. The samples, which were measured for ALT, AST, LDL cholesterol, and total cholesterol, were sent to the Guangdong Laboratory Animals Monitoring Institute for analysis.

### Statistical analysis

In this study, GraphPad Prism was used to analyze the data. To determine significant differences in precise editing efficiency between groups, we utilized unpaired Student's *t*-test (*P < 0.05, **P < 0.01, ***P < 0.001, ****P < 0.0001, and ns indicating not significant). The error bar of each column represents the standard deviation.

### Availability of Data and Materials

All data supporting the findings in this study are available within this Article and its additional files. The whole genome sequencing data and amplicon sequencing data have been deposited in the Genome Sequence Archive of the Beijing Institute of Genomics (BIG) Data Center under BioProject PRJCA022900 (https://ngdc.cncb.ac.cn/bioproject/browse/PRJCA022900) [29]. All other source data generated during this study are available from the corresponding authors upon reasonable request.

### Supplementary Information

**Additional file 1: Figures S1-S11.** With figure legends for Enhancing prime editor flexibility with coiled-coil heterodimers.

**Additional file 2: Table S1-S4.** For Enhancing prime editor flexibility with coiled-coil heterodimers.

**Additional file 3.** Review history.
