# Supplementary material for: Enhancing prime editor flexibility with coiled-coil heterodimers
Source: Genome Biol. 2024 Apr 26;25:108. doi: 10.1186/s13059-024-03257-z (PMC11046888; doi:10.1186/s13059-024-03257-z)
Supplement: Supplementary file 1 — Additional file 1: Figures S1-S11. With figure legends for Enhancing prime editor flexibility with coiled-coil heterodimers. [file 13059_2024_3257_MOESM1_ESM.docx]

**Fig. S1.** Evaluations of prime editing efficiency for intact PE, CC-PE and other split strategies. Summary of intact PE, sPE-P3P4, sPE-N5N6, sPE-ctrl, Intein-PE, MS2-PE, and SunTag-PE editing efficiencies for a total of 6 loci in HEK293T cells. Statistical significance was determined via unpaired *t*-tests (**P* < 0.05, ***P* < 0.01, ****P* < 0.001, *****P* < 0.0001, ns indicating not significant). Error bars indicated the mean ± standard deviation of at least three independent biological replicates.


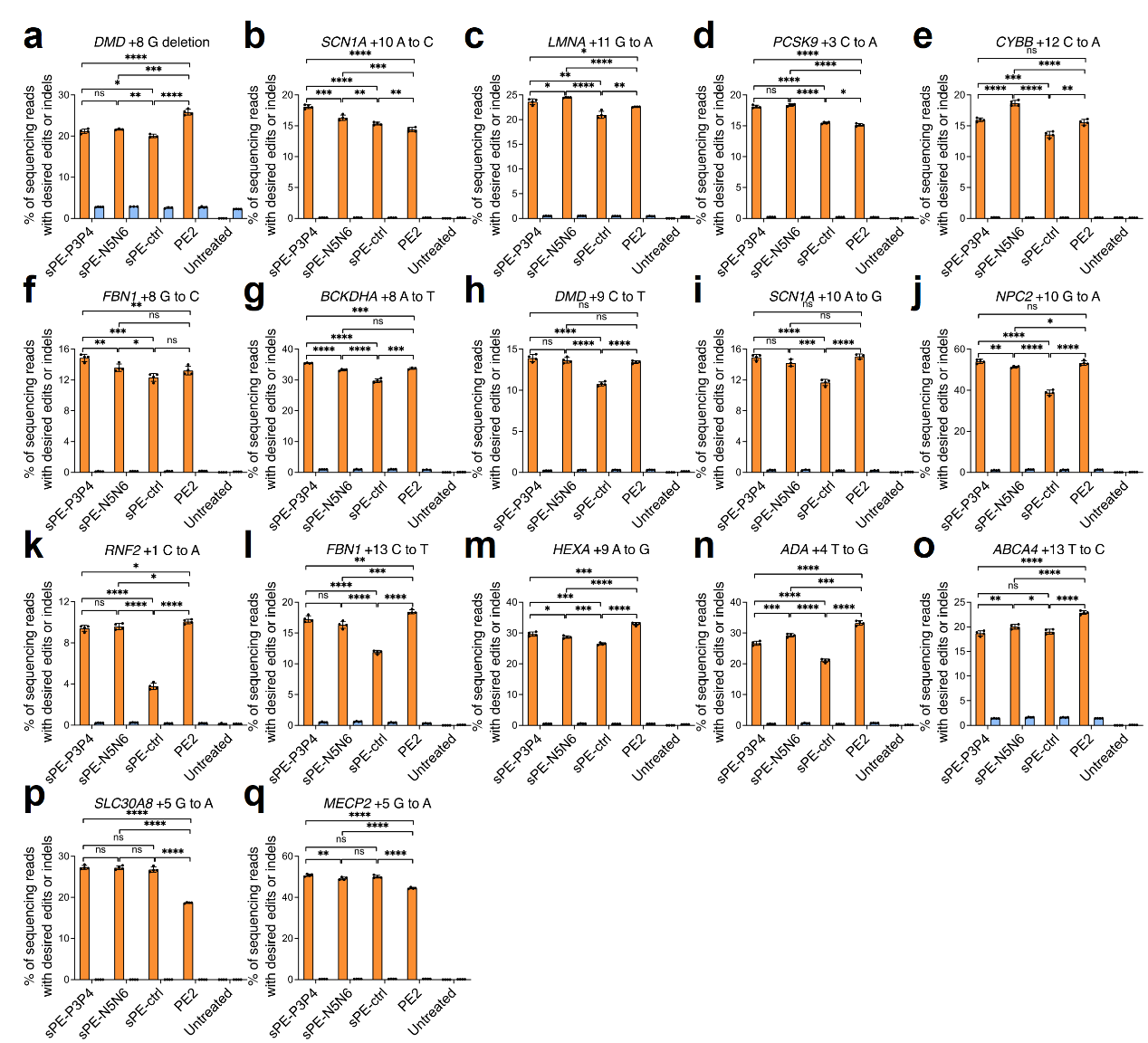


**Fig. S2.** CC-PE enables precise genome editing at various sites in HEK293T cells. **a** Precise base deletion and undesired indel efficiencies of sPE-P3P4, sPE-N5N6, sPE-ctrl, and PE2 at *DMD* (+8 G deletion) locus. **b**-**q** Precise base substitution and undesired indel efficiencies of sPE-P3P4, sPE-N5N6, sPE-ctrl, and PE2 among 16 tested loci. Statistical significance in a-q was determined via unpaired *t*-tests (**P* < 0.05, ***P* < 0.01, ****P* < 0.001, *****P* < 0.0001, ns indicating not significant). Error bars indicated the mean ± standard deviation of at least three independent biological replicates.

**Fig. S3.** Summary of intact PE, sPE-P3P4, sPE-N5N6, and sPE-ctrl undesired indel efficiencies for a total of 32 loci in HEK293T cells. Statistical significance was determined via unpaired *t*-tests (**P* < 0.05, ***P* < 0.01, ****P* < 0.001, *****P* < 0.0001, ns indicating not significant). Error bars indicated the mean ± standard deviation of multiple independent biological replicates.


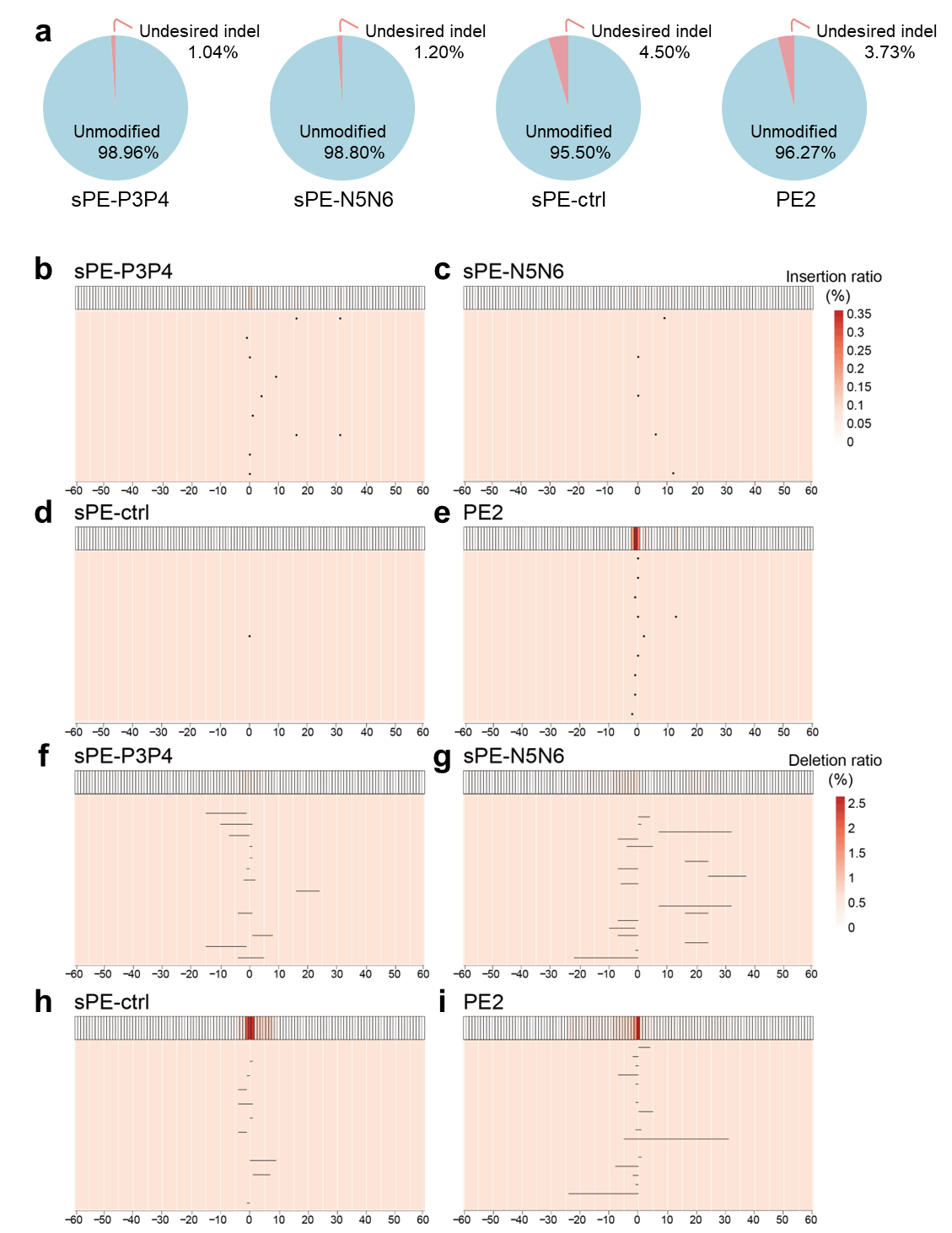


**Fig. S4.** Undesired insertion and deletion efficiencies of CC-PE and PE2 at *HEK3* (+1 T to A) locus. **a** Pie charts representing overall undesired indel formation of sPE-P3P4, sPE-N5N6, sPE-ctrl and PE2 at *HEK3* (+1 T to A) locus. **b**-**e** Undesired insertion ratio and distribution of sPE-P3P4 (**b**), sPE-N5N6 (**c**), sPE-ctrl (**d**) and PE2 (**e**) at *HEK3* locus. **f**-**i** Undesired deletion ratio and length range of sPE-P3P4 (**f**), sPE-N5N6 (**g**), sPE-ctrl (**h**) and PE2 (**i**) at *HEK3* locus. Black lines represent deletion events.


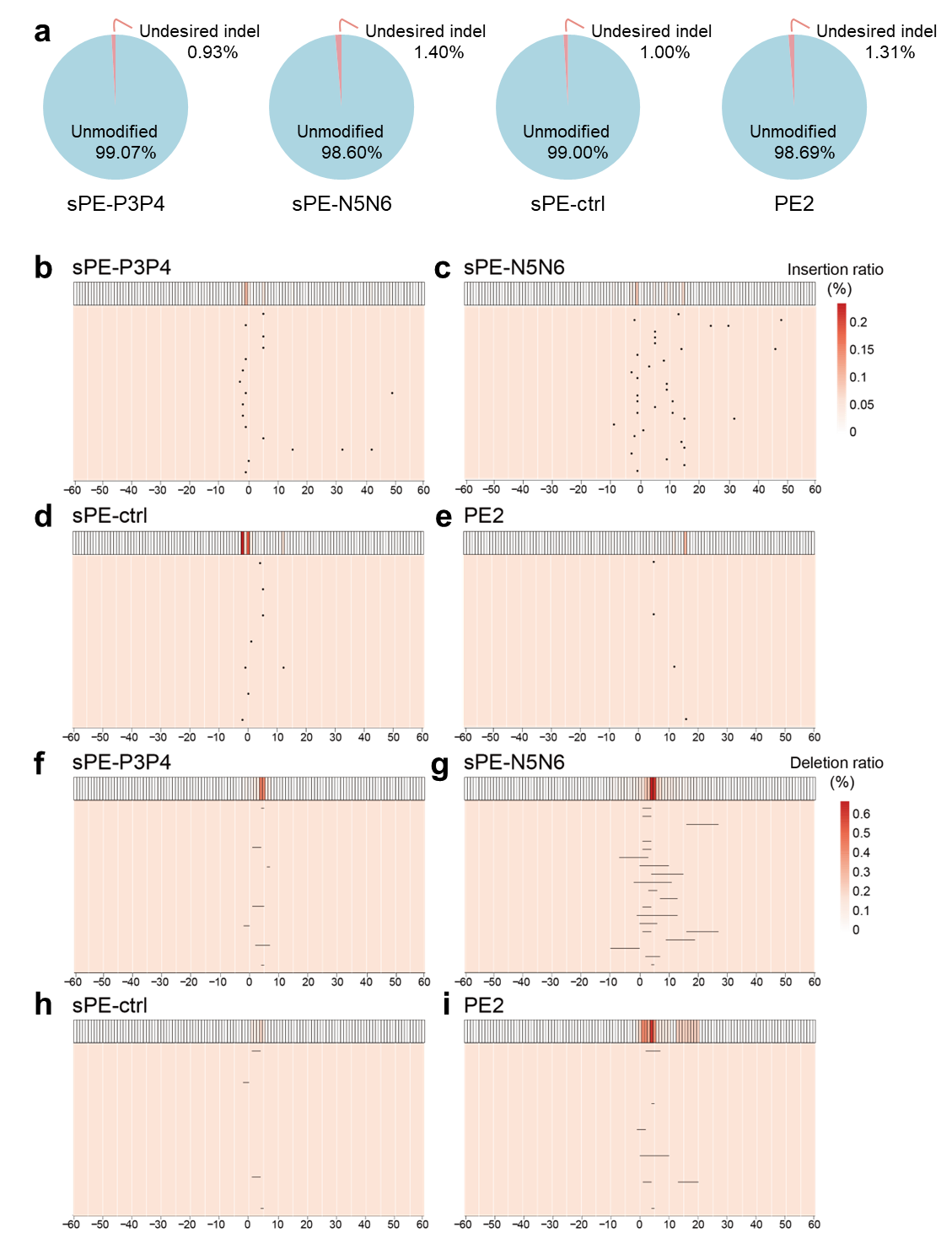


**Fig. S5.** Undesired insertion and deletion efficiencies of CC-PE and PE2 at *B2M* (+1 GAAT deletion) locus. **a** Pie charts representing overall undesired indel formation of sPE-P3P4, sPE-N5N6, sPE-ctrl and PE2 at *B2M* (+1 GAAT deletion) locus. **b**-**e** Undesired insertion ratio and distribution of sPE-P3P4 (**b**), sPE-N5N6 (**c**), sPE-ctrl (**d**) and PE2 (**e**) at *B2M* locus. **f**-**i** Undesired deletion ratio and length range of sPE-P3P4 (**f**), sPE-N5N6 (**g**), sPE-ctrl (**h**) and PE2 (**i**) at *B2M* locus. Black lines represent deletion events.


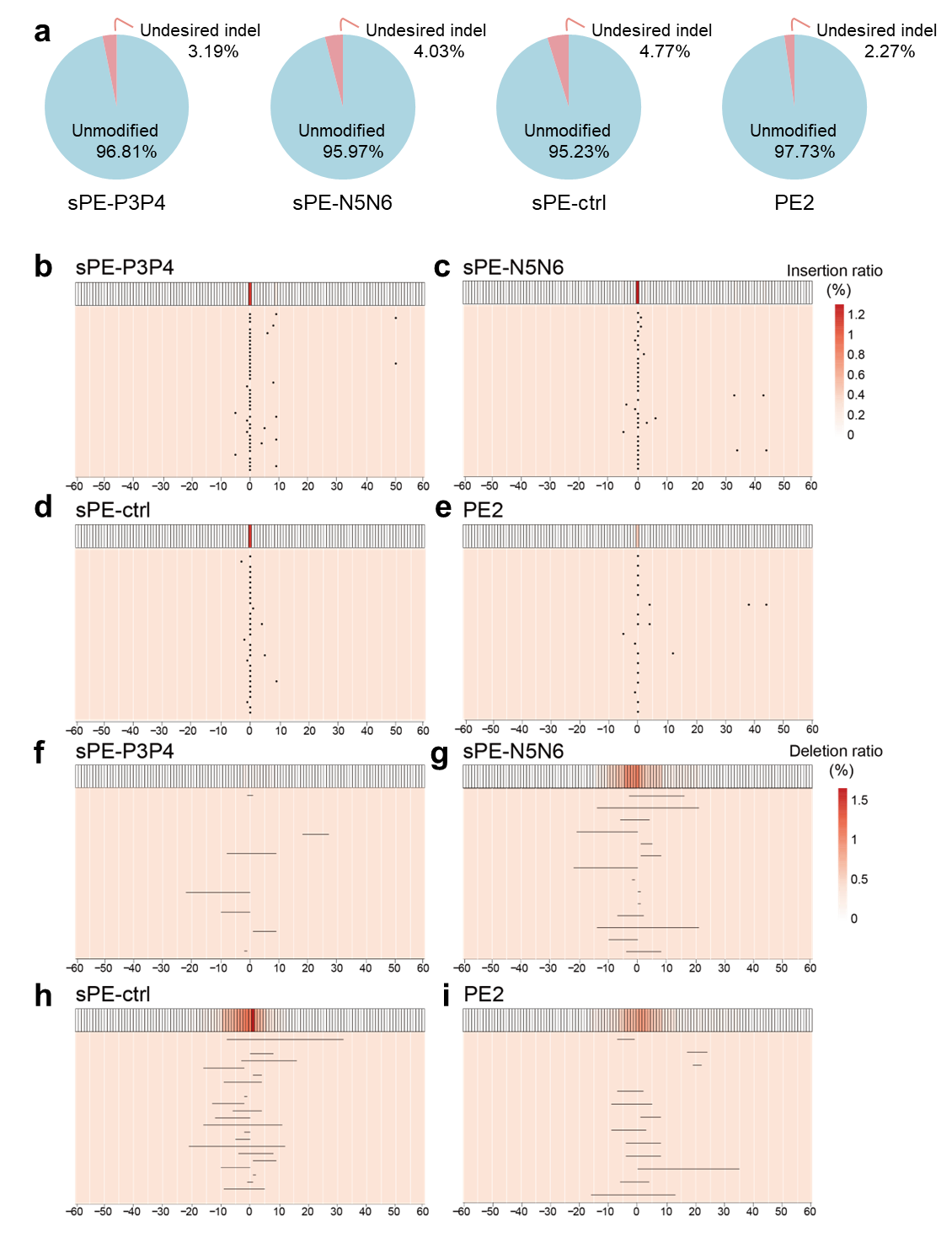


**Fig. S6.** Undesired insertion and deletion efficiencies of CC-PE and PE2 at *VEGFA* (+1 C insertion) locus. **a** Pie charts representing overall undesired indel formation of sPE-P3P4, sPE-N5N6, sPE-ctrl and PE2 at *VEGFA* (+1 C insertion) locus. **b**-**e** Undesired insertion ratio and distribution of sPE-P3P4 (**b**), sPE-N5N6 (**c**), sPE-ctrl (**d**) and PE2 (**e**) at *VEGFA* locus. **f**-**i** Undesired deletion ratio and length range of sPE-P3P4 (**f**), sPE-N5N6 (**g**), sPE-ctrl (**h**) and PE2 (**i**) at *VEGFA* locus. Black lines represent deletion events.


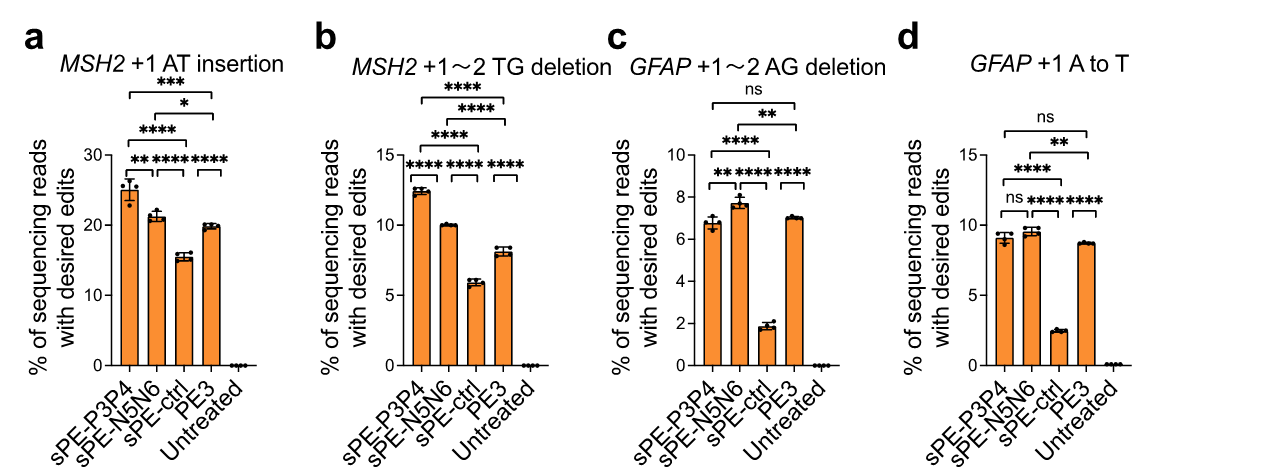


**Fig. S7.** CC-PE enables precise genome editing with the PE3 strategy at various sites in HEK293T cells. **a-d** Precise editing efficiencies of sPE-P3P4, sPE-N5N6, sPE-ctrl, and PE3 at *MSH2* (+1 AT insertion) (**a**), *MSH2* (+1~2 TG deletion) (**b**), *GFAP* (+1~2 AG deletion) (**c**), and *GFAP* (+1 A to T) (**d**) loci. Statistical significance in **a**-**d** was determined via unpaired *t*-tests (**P* < 0.05, ***P* < 0.01, ****P* < 0.001, *****P* < 0.0001, ns indicating not significant). Error bars indicated the mean ± standard deviation of at least three independent biological replicates.


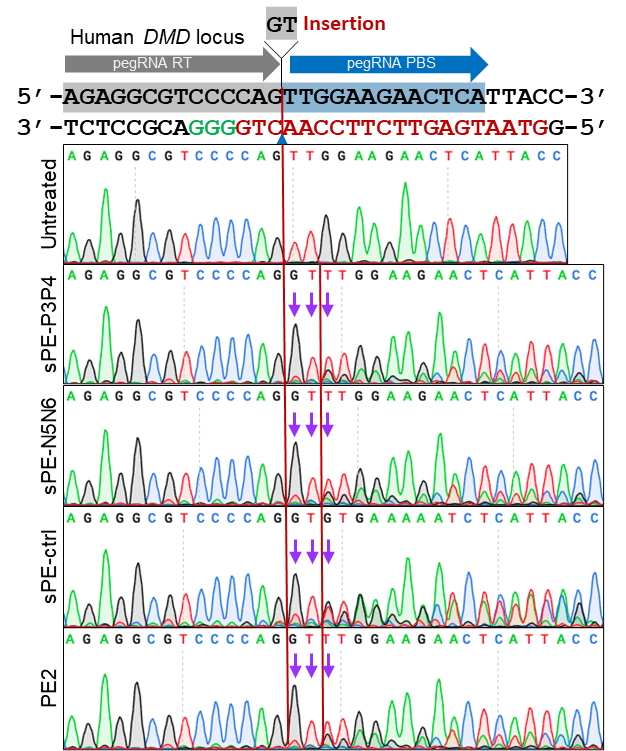


**Fig. S8.** Sanger-sequencing chromatograms of sPE-P3P4, sPE-N5N6, sPE-ctrl, and PE2 harboring the desired insertion at *DMD* (+1 AC insertion) locus in human HEK293T cells. The purple arrow denotes the desired insertion. The *DMD*-specific sgRNA is indicated in red and the PAM sequence is indicated in green.


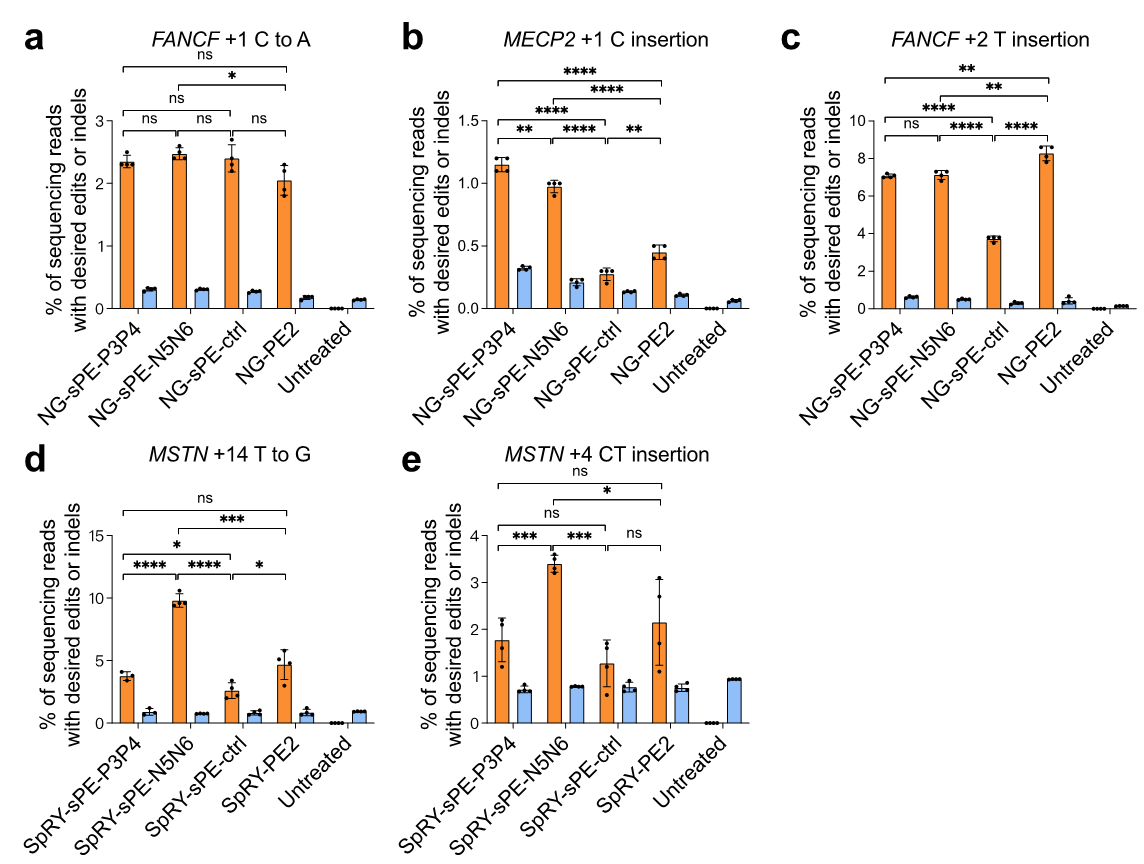


**Fig. S9.** Prime editing using NG-CC-PE or SpRY-CC-PE system. **a-c** Precise editing and undesired indel efficiencies of NG-sPE-P3P4, NG-sPE-N5N6, NG-sPE-ctrl, and NG-PE2 at *FANCF* (+1 C to A) (**a**), *MECP2* (+1 C insertion) (**b**), and *FANCF* (+2 T insertion) (**c**) loci in HEK293T cells. **d-e**. Precise editing and undesired indel efficiencies of SpRY-sPE-P3P4, SpRY-sPE-N5N6, SpRY-sPE-ctrl, and SpRY-PE2 at *MSTN* (+14 T to G) (**d**) and *MSTN* (+4 CT insertion) (**e**) loci in HEK293T cells. Data in **a** and **b** were obtained from three or more independent biological replicates (mean ± s.d.) via unpaired t-tests (**P* < 0.05, ***P* < 0.01, ****P* < 0.001, *****P* < 0.0001, ns indicating not significant).


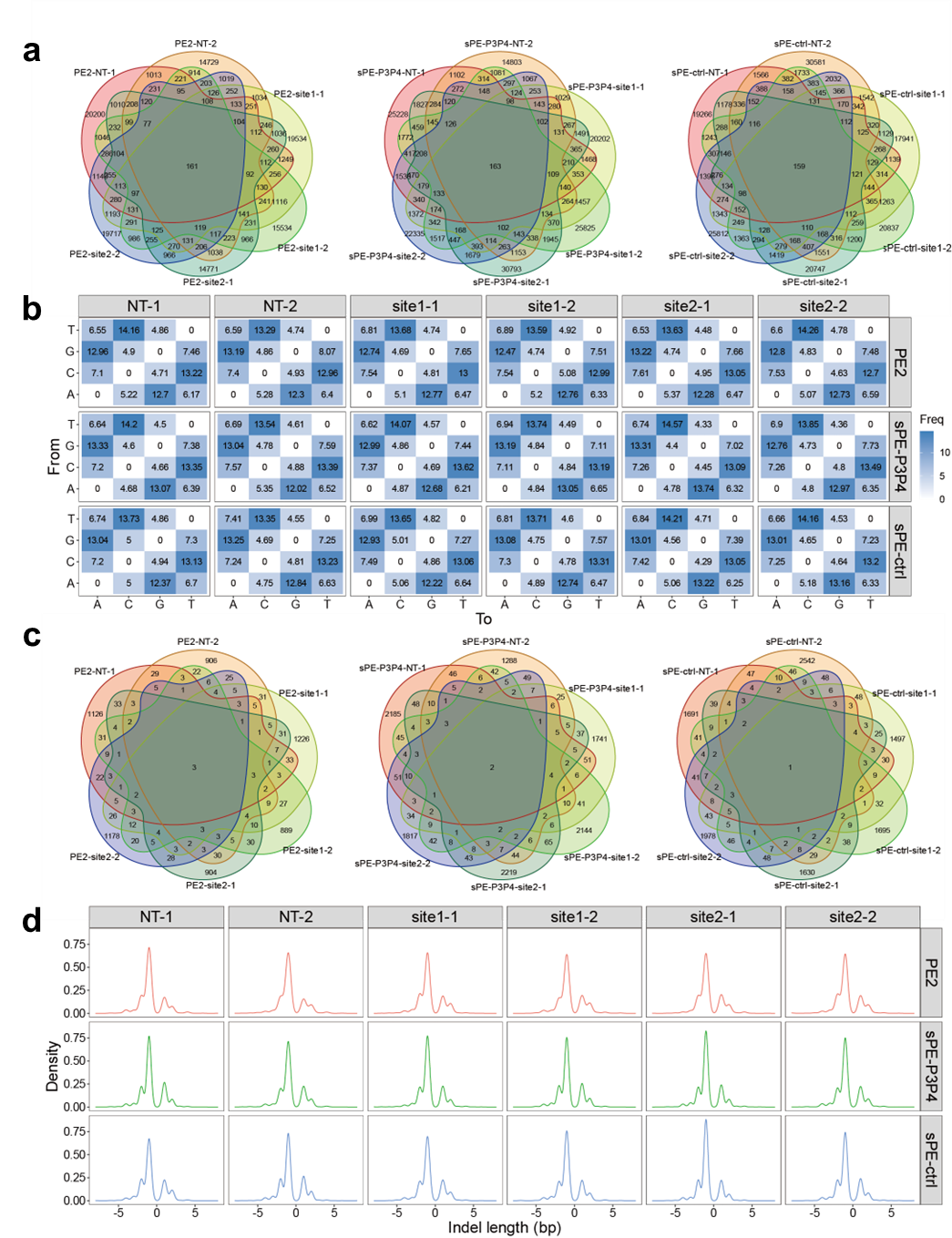


**Fig. S10.** Analysis of mutation characteristics of SNVs and Indels. **a** Venn diagram shows the number of SNVs detected in each sample and its replicates. **b** Heat map shows the proportion of base mutation types of SNV in each sample and its replicates. **c** Venn diagram showing the number of indels detected in each sample and its replicates. **d** The density plot shows the distribution of indel lengths in each sample and its replicates.


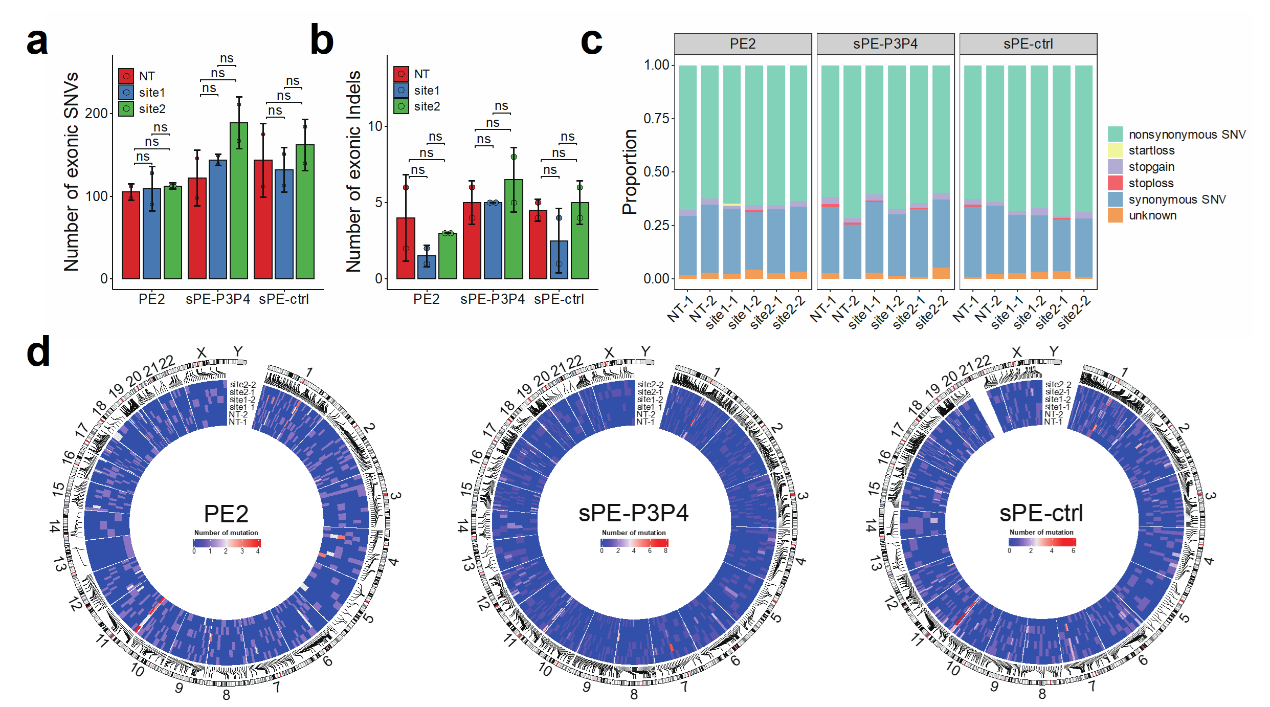


**Fig. S11.** Evaluation of the distribution of genome-wide variation in gene functional regions. **a** The number of SNVs distributed on exons. **b** The number of Indels distributed on exons. **c** Changes in amino acids caused by SNVs on exons. **d** Circosplot shows the genome-wide distribution of exonic SNVs in PE2 (left), sPE-P3P4 (middle), and sPE-ctrl (right). The color represents mutation density within the 1 × 10^7^ bp windows.
