# Supplementary material for: Enhancing prime editor flexibility with coiled-coil heterodimers
Source: Genome Biol. 2024 Apr 26;25:108. doi: 10.1186/s13059-024-03257-z (PMC11046888; doi:10.1186/s13059-024-03257-z)
Supplement: Supplementary file 3 — Additional file 3. Review history. [file 13059_2024_3257_MOESM3_ESM.docx]

**Review History**

**First round of review**

**Reviewer #1: Summary**

In this paper, the authors developed a prime editor connected with a coiled-coil heterodimer (CC-PE) and demonstrated that effective gene editing is possible for human-derived cell line. This simple methodology shows the possibility of inducing useful prime editing in living organisms through delivery vehicles such as AAV viruses that have size limitations in the future. However, it has not been fully verified experimentally. The present form is insufficient for publication in genome biology, and we suggest the following additional experiments.

**Major**

1. When using the prime editor connected to the coiled-coil heterodimer (CC-PE), the efficiency did not seem to increase significantly compared to the existing prime editing (PE2), and only the sites where prime editing was well induced were compared. We propose a comparative analysis of the efficiency of conventional prime editing (PE2) and coiled-coil heterodimer prime editor (CC-PE) on at least 40 target sites, including target sites with low efficiency.

2. In order to show that editing is robustly induced in various human-derived cell lines, we propose to show it in various human-derived cell lines using the Prime editor coiled-coil heterodimer (CC-PE).

3. When using Prime editor (CC-PE) connected coiled-coil heterodimer, off-target properties were not identified. Compared to the existing prime editing (PE2), off-target properties should be compared and analyzed.

**Minor**

1. Why do many indels appear in the PE2 method, which induces single nicking in the target gene? We propose to compare and analyze the efficiency of the existing prime editing (PE2) and the coiled-coil heterodimer prime editor (CC-PE) with the PE3 method.

Reviewer #2: Review of "Enhancing prime editor flexibility with coiled-coil heterodimer"

**Brief summary**

The authors present a convincing argument highlighting the current limitations of prime editing (PE) tools for in vivo applications, arising from the constrained packaging size of adeno-associated viruses (AAV). Subsequently, the authors provide an overview of recent advancements in PE variants designed to overcome these size constraints. These versions include split inteins, MS2, SunTag, and the direct split PE (sPE) system, along with an insight into their limitations that include complicated engineering or reduced editing efficiency.

The authors introduce a simply engineered system as an effective solution. They introduce a coiled-coil dimerization system, that recruits the reverse transcriptase (RT) to the Cas9 nickase (nCas9) using electrostatic and hydrophobic interactions. They fuse two coiled-coil peptides, the P3-P4 pair or the N5-N6 pair, referred to as sPE-P3P4 and sPE-N5N6 respectively. Specifically, P3 or N5 is fused to the C-terminus of nCas9, while P4 or N6 is fused to the N-terminus of RT.

To test their hypothesis, the authors transfected HEK293T cells with sPE-P3P4 and sPE-N5N6 constructs, along with one of three single-guide RNAs (sgRNAs) targeting endogenous loci, B2M (+1 GAAT deletion), HEK3 (+1 T to A), or VEGFA (+1 C insertion). Assessment of on-target endogenous editing was tested using Deep and Sanger sequencing of genomic GNA.

The results demonstrate the success of sPE-P3P4 and sPE-N5N6 in generating the desired specific endogenous edits. These constructs showed higher editing efficiency compared to sPE-ctrl and exhibited similar editing efficiency to PE2. Additionally, the authors examined the rate of off-target indels introduced by their PE systems. At the HEK3 (+1 T to A) locus, sPE-P3P4 and sPE-N5N6 showed lower indel rates compared to PE2. However, at the B2M (+1 GAAT deletion) and VEGFA (+1 C insertion) loci, indel rates were similar to those of PE2 and sPE. Overall, the manuscript presents a solution for the co-delivery of the prime editor nCas9 and the RT, overcoming the size limitation imposed by the AAV packaging limit. Nevertheless, it is critical that this tool undergoes in vivo testing to prove its ability to yield the intended outcomes and confirm its practical use in assisting in vivo prime editing.

Specific comments:

1. The authors only tested the editing efficiency of sPE-P3P4 and sPE-N5N6 in one cell line, HEK293Ts. How does the editing efficiency compare to sPE-ctrl and PE2 in other cell lines at those same genomic loci (B2M (+1 GAAT deletion), HEK3 (+1 T to A), and VEGFA (+1 C insertion)).

2. The authors examined the capacity of sPE-P3P4 and sPE-N5N6 to generate deletions, transition mutations, and insertions in 3 genes only. To more strongly support their statement that sPE-P3P4 and sPE-N5N6 show "superior capabilities than PE2 in precise editing" the authors should provide experimental data showing this trend across at least two more genes for each edit type (3 deletion, 3 insertions and 3 base substitutions). Moreover, when the authors examined the rate of undesired indels at the different genomic loci, their results demonstrated variability between the indel rates at the different sites, indicating that there is need for testing both on-target and off-target editing at more genomic sites/more genes.

3.The authors argue that the current version of prime editors that were designed to overcome the limitations associated with the large size of PE and its inability to be packaged into AAVs include split inteins, MS2, and SunTag and direct split PE (sPE) system. However, the study only compares their novel version of PE with the sPE system. How do sPE-P3P4 and sPE-N5N6 compare to split inteins, MS2, and SunTag PEs?

4. Another limitation of the study is the use of a prime editor with the need for an NGG protospacer adjacent motif (PAM). The authors are encouraged to also test that sPE-P3P4 and sPE-N5N6 works with more flexible PAMs, which increases the number of accessible mutations across the genome. These include spCas9-NG PE for NG PAM recognition and/or SpRY PE variant for unconstrained PAM recognition. This should be a feasible experiment as the authors are testing editing at only a few genomic loci.

5. In figure 1A, it would be helpful to also add an illustration of the unsplit PE2, although it is more established in the field, however, it is one of the main versions of PE that the authors compare their new version to.

6. In figure 2C the authors show the deletion events associated with each editor and demonstrate the length of the deletion using black lines. It is confusing why the authors did not demonstrate a similar full plot for the insertions in figure 2B.

7. A major limitation of the study is the lack of in vivo experiments. The prime focus of the study is to facilitate in vivo delivery of PE using AAVs. Therefore, the strongest validation that their PE versions yield the intended results would be to show experimentally that it can be delivered in vivo to adult mice, and that they yield similar editing efficiency and indel ratios as seen in cells. In vivo PE has been demonstrated broadly; thus, these experiments are feasible.

**Authors Response**

**Point-by-point responses to the reviewers’ comments:**

Reviewer reports:

Reviewer #1: Summary

In this paper, the authors developed a prime editor connected with a coiled-coil heterodimer (CC-PE) and demonstrated that effective gene editing is possible for human-derived cell line. This simple methodology shows the possibility of inducing useful prime editing in living organisms through delivery vehicles such as AAV viruses that have size limitations in the future. However, it has not been fully verified experimentally. The present form is insufficient for publication in genome biology, and we suggest the following additional experiments.

**Response:** We appreciate the positive and encouraging comments from the reviewer. We have studied the comments carefully and revised the manuscript as the reviewer suggested. Our specific responses are presented below.

Major

1. When using the prime editor connected to the coiled-coil heterodimer(CC-PE), the efficiency did not seem to increase significantly compared to the existing prime editing (PE2), and only the sites where prime editing was well induced were compared. We propose a comparative analysis of the efficiency of conventional prime editing (PE2) and coiled-coil heterodimer prime editor (CC-PE) on at least 40 target sites, including target sites with low efficiency.

**Response:** Thanks for the great proposal of comparative analysis of the efficiencies of conventional prime editing and coiled-coil heterodimer prime editor. We have provided a total of 54 target sites, including 32 target sites for PE2 and 22 target sites for PE3. The results are shown in Figure 1, Figure 2, Figure S2, and Figure S7. Compared with intact PE editor (ranging from 2.95% to 87.15%), sPE-P3P4 or sPE-N5N6 obtained comparable (22.2%, 12 of 54) or significantly higher (63.0%, 34 of 54) efficiencies. We added two results in the revised manuscript, including “Design of CC-PE and comparative analysis with existing split strategies” and “Evaluation of performance of the coiled-coil heterodimer-mediated split strategy on the PE3 editor”.

2. In order to show that editing is robustly induced in various human-derived cell lines, we propose to show it in various human-derived cell lines using the Prime editor coiled-coil heterodimer(CC-PE).

**Response:** We couldn't agree more with this good suggestion. We explored the performance of CC-PE in other human cell lines (A549, HCT116, and U2OS), and found that CC-PE could achieve similar performance in other human cell lines. We added following results in the revised manuscript:

“**Efficient precise editing by CC-PE in multiple mammalian cell lines**

The findings above showed that CC-PE had similar or even superior efficiencies to unsplit PE at various genomic loci in HEK293T cells. We next examined the universality of CC-PE in other human cell lines. The editing activities of sPE-P3P4, sPE-N5N6, sPE-ctrl, and unsplit PE were examined at six genomic loci in each of the three cell lines including A549, HCT116, and U2OS cells (Fig. 3a-c). In A549 cells, both sPE-P3P4 and sPE-N5N6 resulted in higher editing efficiencies compared to sPE-ctrl and similar efficiencies to intact PE at *B2M* (+1~4 GAAT deletion) and *HEK3* (+1 A insertion) loci (Fig. 3a). Additionally, significantly increased efficiencies of sPE-P3P4 or sPE-N5N6 compared to sPE-ctrl and unsplit PE were observed at *HEK3* (+1 T to A), *VEGFA* (+1 T deletion and +1 C insertion), and *DMD* (+1 AC insertion) loci (Fig. 3a). In HCT116 cells, sPE-P3P4 and sPE-N5N6 resulted in higher editing efficiencies compared to sPE-ctrl and similar efficiencies to intact PE at *NPC2* (+10 G to A), *VEGFA* (+1 C insertion), and *DMD* (+1 AC insertion) loci (Fig. 3b). The efficiencies of sPE-P3P4 and sPE-N5N6 were significantly higher than those of sPE-ctrl and unsplit PE at *ERCC2* (+12 G to C), *DNMT1* (+1~15 ACAGGGGTGACGGGA deletion), and *EMX1* (+1~15 AGAAGGGCTCCCATC deletion) (Fig. 3b). In U2OS cells, the sPE-P3P4 and sPE-N5N6 achieved significantly increased efficiencies compared to sPE-ctrl and unsplit PE at *ERCC2* (+12 G to C), *NPC2* (+10 G to A), and *DMD* (+1 AC insertion) loci (Fig. 3c). The sPE-P3P4 and sPE-N5N6 exhibited significantly increased efficiencies compared to unsplit PE and similar efficiencies to sPE-ctrl at *VEGFA* (+1 C insertion) locus (Fig. 3c). In addition to the above loci, similar editing efficiencies of CC-PE, sPE-ctrl, and unsplit PE were observed at *EMX1* (+1~15 AGAAGGGCTCCCATC deletion) and *DNMT1* (+1~15 ACAGGGGTGACGGGA deletion) loci (Fig. 3c).

In general, across all 18 edits at eight endogenous genes, both sPE-P3P4 and sPE-N5N6 exhibited superior efficiencies to sPE-ctrl at 15 edits and demonstrated similar or enhanced precise editing activities than that of unsplit PE across all tested loci. These data suggest that the editing efficiency of CC-PE typically surpasses that of direct split PE system and is comparable or even superior to that of intact PE system across various cell lines.”

3. When using Prime editor (CC-PE) connected coiled-coil heterodimer, off-target properties were not identified. Compared to the existing prime editing (PE2), off-target properties should be compared and analyzed.

**Response:** Thank you for the kind remind to analyze and compare off-target properties among CC-PE, sPE-ctrl, and the existing prime editing (PE2). We performed whole-genome sequencing (WGS) to identify the off-target properties of CC-PE, sPE-ctrl, and PE2. Detailed results were appended to the revised manuscript as follows:

“**Bioinformatic analysis of pegRNA-independent off-target effects of CC-PE through whole-genome sequencing**

Next, we performed whole-genome sequencing (WGS) on HEK293T cells to assess pegRNA-independent off-target effects of CC-PE at the genome-wide level. Due to the comparable precise editing efficiencies of sPE-P3P4 and sPE-N5N6 at all tested loci, sPE-P3P4 was selected as the representative for further off-target analysis. First, we assessed whether more mutations would be generated with the pegRNA of target site (site1, *B2M* +1~4 deletion; site2, *HEK3* +1 T to A) than that of the pegRNA of non-target site (NT). WGS results revealed that there was no significant difference in the number of single nucleotide variants (SNVs) and indels between the pegRNA of non-targeted site and the pegRNA of targeted site in sPE-P3P4, PE2, and sPE-ctrl (**Fig. 5a, b**). The Venn diagram displayed that very few overlapping mutations occurred across experimental samples and replicates, suggesting that these mutations were spontaneous events and happened randomly in the genome (**Fig. S10a, c**).

We also examined the potential impact of these mutations on gene function. The results showed that there was no difference in the distribution of SNVs and indels on genomic features among samples (**Fig. 5c, d**). Only a small number of mutations resided within exons, and the count of these exon mutations did not present any significant difference across samples (**Fig. S11a, b**). Furthermore, base substitution types of SNVs and lengths of indels were unbiased among samples (**Fig 5e, f; Fig. S10b, d**). For SNVs, the changes of amino acids resulting from mutations showed no difference among samples (**Fig. S11c**). The mutations on genome-wide coordinates revealed by circos plots exhibited that most SNVs were distributed in mutation hotspots, and there were no regional and sample bias in SNVs and indels at the genome-wide level and exon level (**Fig 5g, h; Fig. S11d**). Taken together, these results suggest that these mutations happened spontaneously and randomly, and there is no extra pegRNA-independent off-target caused by CC-PE. This implies that CC-PE could be a practical genome editing tool with manageable mutation handling.”

Minor

1. Why do many indels appear in the PE2 method, which induces single nicking in the target gene? We propose to compare and analyze the efficiency of the existing prime editing (PE2) and the coiled-coil heterodimer prime editor (CC-PE) with the PE3 method.

**Response:** Thanks for the proposal. It has been reported that nCas9 (H840A) used in PE2 method can cleave both strands, producing unwanted DSBs and thus inducing unwanted indels caused by error-prone repair of DSBs (Jaesuk Lee et. al, 2023, Nature Communications), which is the same as many indels appear in the PE2 method at specific sites here. We also tested a total of 22 genomic loci to compare and analyze the efficiency of the existing prime editing (PE2) and CC-PE with the PE3 method. Enhanced prime editing could be further induced by using the CC-PE with the PE3 method. The related results were added as follows:

“**Evaluation of performance of the coiled-coil heterodimer-mediated split strategy on the PE3 editor**

Next, we also evaluate the performance of the coiled-coil heterodimer-mediated split strategy on the PE3 editor, an optimized strategy being able to improve prime editing efficiency, which employs an additional sgRNA to nick the non-edited strand using the Cas9(H840A) nickase already present in PE2, directing DNA repair by using the edited strand as a template [1]. To assess the effectiveness of CC-PE editor with PE3 strategy, we examined frequency of various types of precise edits including insertions, deletions, and substitutions in 22 genomic loci via deep amplicon sequencing. The precise editing efficiencies of CC-PE among these loci ranged from 5.12% to 87.53%, exceeding 50% in 10 genomic loci. (**Fig. 2a, b**). The sPE-P3P4 and sPE-N5N6 resulted in significantly higher efficiencies of precise edits than that of direct split PE at all tested loci except for *RNF2* (+6 G to A), *VEGFA* (+5 G to T), and *PRNP* (+6 G to T) loci (**Fig. 2a****; Fig. S7**). Additionally, they displayed significantly higher efficiencies at 14 out of 22 loci and showed no significant difference at 7 out of 22 loci when compared to unsplit PE3 (**Fig. 2a; Fig. S7**). The sPE-P3P4 and sPE-N5N6 exhibited stronger precise editing activities than both unsplit PE3 and sPE-ctrl at 13 out of 22 (59.1%) tested loci (**Fig. 2a; Fig. S7**). Specifically, as an example, we noted that when the *DMD* locus, a therapeutically relevant gene, was used as the gene of interest, the precise insertion efficiencies of both sPE-P3P4 and sPE-N5N6 were comparable to intact PE3, while significantly superior to that of sPE-ctrl (**Fig. 2a; Fig. S8**). These results reaffirmed that CC-PE editor outperforms direct split PE when combined with the PE3 strategy, while maintaining or exceeding the capabilities of unsplit PE3.”

Reviewer #2: Review of "Enhancing prime editor flexibility with coiled-coil heterodimer"

Brief summary

The authors present a convincing argument highlighting the current limitations of prime editing (PE) tools for in vivo applications, arising from the constrained packaging size of adeno-associated viruses (AAV). Subsequently, the authors provide an overview of recent advancements in PE variants designed to overcome these size constraints. These versions include split inteins, MS2, SunTag, and the direct split PE (sPE) system, along with an insight into their limitations that include complicated engineering or reduced editing efficiency.

The authors introduce a simply engineered system as an effective solution. They introduce a coiled-coil dimerization system, that recruits the reverse transcriptase (RT) to the Cas9 nickase (nCas9) using electrostatic and hydrophobic interactions. They fuse two coiled-coil peptides, the P3-P4 pair or the N5-N6 pair, referred to as sPE-P3P4 and sPE-N5N6 respectively. Specifically, P3 or N5 is fused to the C-terminus of nCas9, while P4 or N6 is fused to the N-terminus of RT.

To test their hypothesis, the authors transfected HEK293T cells with sPE-P3P4 and sPE-N5N6 constructs, along with one of three single-guide RNAs (sgRNAs) targeting endogenous loci, B2M (+1 GAAT deletion), HEK3 (+1 T to A), or VEGFA (+1 C insertion). Assessment of on-target endogenous editing was tested using Deep and Sanger sequencing of genomic GNA.

The results demonstrate the success of sPE-P3P4 and sPE-N5N6 in generating the desired specific endogenous edits. These constructs showed higher editing efficiency compared to sPE-ctrl and exhibited similar editing efficiency to PE2. Additionally, the authors examined the rate of off-target indels introduced by their PE systems. At the HEK3 (+1 T to A) locus, sPE-P3P4 and sPE-N5N6 showed lower indel rates compared to PE2. However, at the B2M (+1 GAAT deletion) and VEGFA (+1 C insertion) loci, indel rates were similar to those of PE2 and sPE. Overall, the manuscript presents a solution for the co-delivery of the prime editor nCas9 and the RT, overcoming the size limitation imposed by the AAV packaging limit. Nevertheless, it is critical that this tool undergoes in vivo testing to prove its ability to yield the intended outcomes and confirm its practical use in assisting in vivo prime editing.

**Response:** We appreciated for your valuable comments and suggestions. We have performed *in vivo* prime editing experiments by using dual AAV to deliver CC-PE, which proved its ability to yield the intended outcomes and confirmed its practical use in assisting *in vivo* precise editing.

Specific comments:

1.	The authors only tested the editing efficiency of sPE-P3P4 and sPE-N5N6 in one cell line, HEK293Ts. How does the editing efficiency compare to sPE-ctrl and PE2 in other cell lines at those same genomic loci (B2M (+1 GAAT deletion), HEK3 (+1 T to A), and VEGFA (+1 C insertion)).

**Response:** We couldn't agree more with this good suggestion. We explored the performance of CC-PE in other human cell lines (A549, HCT116, and U2OS), and found that CC-PE could achieve similar performance in other human cell lines. We added following results in the revised manuscript:

“**Efficient precise editing by CC-PE in multiple mammalian cell lines**

The findings above showed that CC-PE had similar or even superior efficiencies to unsplit PE at various genomic loci in HEK293T cells. We next examined the universality of CC-PE in other human cell lines. The editing activities of sPE-P3P4, sPE-N5N6, sPE-ctrl, and unsplit PE were examined at six genomic loci in each of the three cell lines including A549, HCT116, and U2OS cells (Fig. 3a-c). In A549 cells, both sPE-P3P4 and sPE-N5N6 resulted in higher editing efficiencies compared to sPE-ctrl and similar efficiencies to intact PE at *B2M* (+1~4 GAAT deletion) and *HEK3* (+1 A insertion) loci (Fig. 3a). Additionally, significantly increased efficiencies of sPE-P3P4 or sPE-N5N6 compared to sPE-ctrl and unsplit PE were observed at *HEK3* (+1 T to A), *VEGFA* (+1 T deletion and +1 C insertion), and *DMD* (+1 AC insertion) loci (Fig. 3a). In HCT116 cells, sPE-P3P4 and sPE-N5N6 resulted in higher editing efficiencies compared to sPE-ctrl and similar efficiencies to intact PE at *NPC2* (+10 G to A), *VEGFA* (+1 C insertion), and *DMD* (+1 AC insertion) loci (Fig. 3b). The efficiencies of sPE-P3P4 and sPE-N5N6 were significantly higher than those of sPE-ctrl and unsplit PE at *ERCC2* (+12 G to C), *DNMT1* (+1~15 ACAGGGGTGACGGGA deletion), and *EMX1* (+1~15 AGAAGGGCTCCCATC deletion) (Fig. 3b). In U2OS cells, the sPE-P3P4 and sPE-N5N6 achieved significantly increased efficiencies compared to sPE-ctrl and unsplit PE at *ERCC2* (+12 G to C), *NPC2* (+10 G to A), and *DMD* (+1 AC insertion) loci (Fig. 3c). The sPE-P3P4 and sPE-N5N6 exhibited significantly increased efficiencies compared to unsplit PE and similar efficiencies to sPE-ctrl at *VEGFA* (+1 C insertion) locus (Fig. 3c). In addition to the above loci, similar editing efficiencies of CC-PE, sPE-ctrl, and unsplit PE were observed at *EMX1* (+1~15 AGAAGGGCTCCCATC deletion) and *DNMT1* (+1~15 ACAGGGGTGACGGGA deletion) loci (Fig. 3c).

In general, across all 18 edits at eight endogenous genes, both sPE-P3P4 and sPE-N5N6 exhibited superior efficiencies to sPE-ctrl at 15 edits and demonstrated similar or enhanced precise editing activities than that of unsplit PE across all tested loci. These data suggest that the editing efficiency of CC-PE typically surpasses that of direct split PE system and is comparable or even superior to that of intact PE system across various cell lines.”

2.	The authors examined the capacity of sPE-P3P4 and sPE-N5N6 to generate deletions, transition mutations, and insertions in 3 genes only. To more strongly support their statement that sPE-P3P4 and sPE-N5N6 show "superior capabilities than PE2 in precise editing" the authors should provide experimental data showing this trend across at least two more genes for each edit type (3 deletion, 3 insertions and 3 base substitutions). Moreover, when the authors examined the rate of undesired indels at the different genomic loci, their results demonstrated variability between the indel rates at the different sites, indicating that there is need for testing both on-target and off-target editing at more genomic sites/more genes.

**Response:** Thanks for the great suggestion to strengthen our statement. To fully verify the superior capabilities of CC-PE compared with direct split PE and conventional PE, we tested a total of 54 genomic loci, encompassing various types of precise edits including 35 substitutions targeting 28 genes, 12 insertions targeting 12 genes, and 7 deletions targeting 7 genes. As shown in Figure 1 and Figure 2 in revised manuscript, deep amplicon sequencing results, including on-target and undesired indel products, indicate this trend that CC-PE editor outperforms direct split PE, while maintaining or exhibiting superior capabilities compared to conventional PE, and we found that the proportion of undesired indel byproducts of CC-PE was not higher than sPE-ctrl and unsplit PE2 (Fig. S3). Moreover, we performed whole-genome sequencing (WGS) to identify the off-target properties of CC-PE, sPE-ctrl, and PE2 as shown in Figure 5, Figure S10, and Figure S11.

3.	The authors argue that the current version of prime editors that were designed to overcome the limitations associated with the large size of PE and its inability to be packaged into AAVs include split inteins, MS2, and SunTag and direct split PE (sPE) system. However, the study only compares their novel version of PE with the sPE system. How do sPE-P3P4 and sPE-N5N6 compare to split inteins, MS2, and SunTag PEs?

**Response:** Thanks for the valuable advice. Following results are added into revised manuscript “We compared editing efficiency of CC-PE with unsplit PE and 4 previously reported split strategies, including direct split PE (sPE) without affinity modules (sPE-ctrl), Intein-PE, MS2-PE, and SunTag-PE, across six endogenous loci (NF1, EMX1, ERCC2, B2M, HBB, and DNMT1) in HEK293T cells through deep amplicon sequencing. The results showed that CC-PE had increased, or at least, equivalent precise editing efficiencies compared to those of unsplit PE, sPE, Intein-PE, MS2-PE, and SunTag-PE at all six tested endogenous loci (Fig. 1c-h; Fig. S1). We also noted that sPE-ctrl achieved higher or comparable efficiency compared to the other split systems of PE (Fig. S1), thus we chose sPE-ctrl as the control for subsequent studies. Notably, we found that the efficiencies of CC-PE-mediated precise editing were higher than those of sPE-ctrl at all tested endogenous sites and unsplit PE at four endogenous sites (EMX1, ERCC2, HBB, and DNMT1) in HEK293T cells (Fig. 1c-h).”

4.	Another limitation of the study is the use of a prime editor with the need for an NGG protospacer adjacent motif (PAM). The authors are encouraged to also test that sPE-P3P4 and sPE-N5N6 works with more flexible PAMs, which increases the number of accessible mutations across the genome. These include spCas9-NG PE for NG PAM recognition and/or SpRY PE variant for unconstrained PAM recognition. This should be a feasible experiment as the authors are testing editing at only a few genomic loci.

**Response:** Thanks for the practical suggestion. We further designed NG-CC-PE (SpCas9-NG variant for NG PAM: NG-sPE-P3P4 and NG-sPE-N5N6) and SpRY-CC-PE (SpCas9-SpRY variant for NRN PAM: SpRY-sPE-P3P4 and SpRY-sPE-N5N6) to increase the number of accessible mutations across the genome. Overall, across different edits at endogenous genomic loci using NG-CC-PE or SpRY-CC-PE system, CC-PE typically outperformed that of sPE-ctrl (15 of 17 loci) and achieved higher or similar levels of precisely edited efficiencies than unsplit PE2 (16 of 17 loci). Our results demonstrate that the conventional PAM preferences could be relaxed by integrating previously identified SpCas9 variants into the CC-PE editor. Following results are added into revised manuscript:

**“Engineering SpCas9-NG-PE and SpRY-PE with coiled-coil heterodimer for more flexible editing scope**

Conventionally, the targetable regions within the genome using conventional prime editors are greatly limited due to the NGG PAM preference of SpCas9. Subsequent reports showed that integrating evolved Cas9 variants, such as SpCas9-NG with NG PAM and SpCas9-SpRY with NRN PAM, into prime editors conferred them with the capacity to expand the scope of genome editing [2, 3]. Therefore, we proceeded to design two additional types of CC-PE with more flexible PAM: NG-CC-PE (NG-sPE-P3P4 and NG-sPE-N5N6) and SpRY-CC-PE (SpRY-sPE-P3P4 and SpRY-sPE-N5N6), respectively. Accordingly, the pegRNAs were designed to bear NG PAM or NRN PAM, which could introduce diverse types of mutations, including insertions, deletions, and substitutions at target sites.

For SpCas9-NG, a variant that can expand the targeting range by relaxing preferences for the third nucleobase of the PAM [4], we examined the editing efficiencies at 9 NG PAM target sites using the NG-CC-PE system. Increased precise editing efficiencies of NG-sPE-P3P4 and NG-sPE-N5N6 were obtained compared to NG-sPE-ctrl at all tested loci except for *FANCF* (+1 C to A) (**Fig. 4a; Fig. S9a-c**). The precise editing efficiencies of NG-sPE-P3P4 or NG-sPE-N5N6 were comparable to NG-PE2 performance levels at *FANCF* (+1 G deletion), *MECP2* (+1 C deletion), *MECP2* (+1 C to A), and *VEGFA* (+1 T insertion) loci (**Fig. 4a**). Notably, at *VEGFA* (+1 C to A), *MECP2* (+1 A insertion), *FANCF* (+1 C to A), and *MECP2* (+1 C insertion) loci, NG-sPE-P3P4 or NG-sPE-N5N6 had higher average editing efficiencies than NG-PE2 (**Fig. 4a; Fig. S9a, b**). Slightly lower performance of the NG-sPE-P3P4 and NG-sPE-N5N6 compared to NG-PE2 was observed at only one locus (*FANCF*, +2 T insertion) (**Fig. S9c**).

For SpCas9-SpRY, a variant almost entirely removing PAM restriction for genome editing [5], we designed SpRY-CC-PE system and tested its editing efficiency at 8 NRN PAM target loci. The SpRY-sPE-P3P4 and SpRY-sPE-N5N6 resulted in higher efficiencies of precise editing than that of SpRY-sPE-ctrl at all tested loci except for *MSTN* (+4 CT insertion) locus (**Fig. 4b; Fig. S9d, e**). The editing efficiency of SpRY-sPE-P3P4 was comparable to that of SpRY-PE2, while SpRY-sPE-N5N6 outperformed SpRY-PE2 at *DYRK1A* (+5 A deletion), *MSTN* (+1 G deletion), *CUL3* (+1 A to G), *CUL3* (+8 C insertion), *MSTN* (+14 T to G) and *MSTN* (+4 CT insertion) loci (**Fig. 4b; Fig. S9d, e**). Notably, SpRY-sPE-P3P4 and SpRY-sPE-N5N6 achieved higher editing efficiencies than SpRY-PE2 at *CUL3* (+12~13 CA deletion) locus (**Fig. 4b**).

Overall, across different edits at endogenous genomic loci, NG-CC-PE and SpRY-CC-PE system outperformed sPE-ctrl (15 of 17 loci) and achieved higher or similar editing efficiencies than unsplit PE2 (16 of 17 loci). These findings demonstrate that integrating previously identified SpCas9 variants into the CC-PE editor can relax conventional PAM preferences, thereby expanding the scope of spacer sequence selections and potential application of CC-PE with PAM flexibility.”

5.	In figure 1A, it would be helpful to also add an illustration of the unsplit PE2, although it is more established in the field, however, it is one of the main versions of PE that the authors compare their new version to.

**Response:** Thank you for the kind remind. Undoubtedly, unsplit PE2 is more established in the field and one of the main versions of PE that we compare our new version to. The illustrations of the unsplit PE2 and direct split PE were added in Figure 1A in the revised manuscript.

6.	In figure 2C the authors show the deletion events associated with each editor and demonstrate the length of the deletion using black lines. It is confusing why the authors did not demonstrate a similar full plot for the insertions in figure 2B.

**Response:** Thanks for the question. We also demonstrate a similar full plot for the insertions in the Figure S4b-e, Figure S5b-e and Figure S6b-e within the revised manuscript.

7.	A major limitation of the study is the lack of in vivo experiments. The prime focus of the study is to facilitate in vivo delivery of PE using AAVs. Therefore, the strongest validation that their PE versions yield the intended results would be to show experimentally that it can be delivered in vivo to adult mice, and that they yield similar editing efficiency and indel ratios as seen in cells. In vivo PE has been demonstrated broadly; thus, these experiments are feasible.

**Response:** Thank you for the valuable suggestion. We supplemented our study with in vivo experiments. Dual AAV delivery of CC-PE systems demonstrated high efficiency, reaching up to 45.9%, in achieving prime editing of Pcsk9 gene in the mouse liver. We added following results in the revised manuscript:

“**Delivery of CC-PE by dual AAVs for *in vivo* genome editing**

We next verified whether CC-PE could be delivered flexibly and yield precise editing *in vivo* using dual AAVs. To maximize *in vivo* prime editing efficiency, we applied the RNA structural motif, evopreQ_1_, to modify the 3′ terminus of pegRNAs (EpegRNA), a method that has been reported to improve prime editing efficiency by enhancing pegRNA stability and preventing its degradation [6].

Murine proprotein convertase subtilisin/kexin type 9 (*Pcsk9*) was chosen as target gene for *in vivo* verification, due to its potential in treating familial hypercholesterolemia. We designed pegRNAs along with corresponding nicking sgRNAs targeting the first exon of *Pcsk9* gene to insert a stop codon (TGA) or T to abolish Pcsk9 function. Precise editing efficiencies were initially evaluated *in vitro*. Mouse embryonic fibroblasts (MEFs) were co-electroporated with the indicating pegRNA and unsplit/split PE3. Deep sequencing showed that sPE-P3P4 mediated precise insertion efficiencies were significantly higher compared to direct split PE (for TGA insertion: 35.6±1.1 vs 23.4±1.2; for T insertion: 26.3±0.7 vs 21.5±0.6) and unsplit PE3 (for TGA insertion: 35.6±1.1 vs 29.0±0.3; for T insertion: 26.3±0.7 vs 19.0±0.1) (**Fig. 6a, b**). The efficiencies of TGA insertion were overall higher than that of T insertion (**Fig. 6a, b**). We also observed that the modified EpegRNA possess higher prime editing activity (**Fig. 6a, b**). Therefore, sPE-P3P4 and the EpegRNA with TGA insertion were chosen for package into AAV8, which exhibits high affinity for hepatocytes and specifically targets the liver, for disruption of *Pcsk9* gene *in vivo* (**Fig. 6c**). Two AAV vectors were constructed: the first vector contained EF-1a short promoter (EFS)-driven nCas9(H840A) tethered with coiled-coil peptide P3 at C-terminal; the second vector contained EFS-driven RT tethered with coiled-coil peptide P4 at N-terminal, U6 promoter-directed *Pcsk9*-EpegRNA, and corresponding nicking sgRNA expression cassettes (**Fig. 6c**). AAV vectors of the direct split PE system were also constructed as a control (**Fig. 6d**). All vectors were within the 4.8 kb packaging capacity of AAV and were packaged into AAV8 (**Fig. 6c, d**). The dual AAV8 sPE-P3P4 or sPE-ctrl (5 × 10^11^ viral genomes each) were administrated into 2-week-old mice via intraperitoneal injection (*n* = 3 for each group) (**Fig. 6e**). Four weeks after injection, total plasma and livers were harvested from injected and untreated control mice (**Fig. 6e**). Twelve pieces of samples were collected from the liver of each AAV8-injected (*n* = 3 for each group) or untreated (*n* = 2) control mice, and genomic DNA was extracted for further analysis. Sanger sequencing showed that sPE-P3P4 mediated higher prime editing efficiencies compared to sPE-ctrl *in vivo* (**Fig. 6f**). Amplicon deep sequencing further confirmed the successful insertion of TGA stop codon into the *Pcsk9* gene *in vivo* by both sPE-P3P4 and sPE-ctrl (**Fig. 6g**). The average precise insertion efficiencies of sPE-P3P4 (35.8%, *n* = 3; ranging from 25.7% to 45.9%) significantly surpassed that of sPE-ctrl (17.6%, n = 3; ranging from 10.9% to 24.3%) (**Fig. 6g, h**). The average undesired indel efficiencies of sPE-P3P4 and sPE-ctrl were 1.1% (*n* = 3) and 0.7% (*n* = 3), respectively (**Fig. 6i**).

Loss of Pcsk9 leads to reduction in low-density lipoprotein (LDL) cholesterol[7]. Fully in line with Pcsk9 function, both total cholesterol and LDL cholesterol levels significantly reduced in sPE-P3P4-injected mice compared to the untreated controls (**Fig. 6j, k**). Importantly, alanine aminotransferase (ALT) and aspartate transaminase (AST) levels in the injected mice were not elevated, suggesting no apparent hepatocellular injury (**Fig. 6l, m**). Altogether, these data robustly demonstrate that CC-PE can be effectively delivered for *in vivo* precise editing.”

**Second round of review**

Reviewer reports:

Reviewer #1: The authors have conducted additional experiments and I have no further requests for the paper.

**Response:** Thanks for your valuable comments for our revised manuscript.
